# QGFace-LLaVA: Quality-Aware Controlled Fusion of Structured Side Information for Face Analysis Under Imperfect Metadata

**DOI:** 10.3390/biomimetics11080582

**Published:** 2026-08-14

**Authors:** Jinping Feng, Nan Xu, Xi Li, Zhongtao Fu, Zhenhua Xiao, Zhenghua Huang

**Affiliations:** 1 School of Electrical and Information Engineering, Wuhan Institute of Technology, Wuhan 430205, China; 22403010011@stu.wit.edu.cn (J.F.); 06090404@wit.edu.cn (N.X.); 2 College of Information and Artificial Intelligence, Nanchang Institute of Science and Technology, Nanchang 330108, China; zhongtao.fu@wit.edu.cn (Z.F.); xiaozh@hbc.edu.cn (Z.X.); 22403010055@stu.wit.edu.cn (Z.H.)

**Keywords:** multimodal large language models, biomimetic perception, face analysis, structured metadata, quality-aware fusion, metadata reliability

## Abstract

Biomimetic perception systems integrate heterogeneous cues selectively rather than treating all available information as equally reliable. Inspired by this principle, this study proposes QGFace-LLaVA, a multimodal large language model (MLLM)-centered framework for robust face analysis under imperfect metadata. A pretrained MLLM serves as the shared prompt-conditioned visual–language reasoning backbone, while structured side information, including age, gender, confidence cues, and availability indicators, is regulated through task-aware quality estimation, reliability-guided metadata calibration, gated residual correction, and counterfactual metadata reliability regularization (CMRR). Experiments on FER2013, CelebA-40, and UTKFace cover facial expression recognition, facial attribute recognition, and age estimation under clean, noisy, missing, shuffled, naturally erroneous, and counterfactual metadata conditions. The results show that metadata utility depends jointly on task relevance, metadata reliability, and fusion strategy, and that clean-setting gains do not necessarily imply robustness. QGFace-LLaVA reduces harmful dependence on unreliable metadata, while CMRR provides additional stability under corruption and mismatch. Overall, the framework transfers biomimetic selective cue integration into MLLM-based face analysis by treating metadata as reliability-controlled auxiliary evidence rather than a uniformly beneficial input.

## 1. Introduction

Face analysis is an important topic in computer vision, affective computing, and human-centered artificial intelligence because facial images contain rich visual cues related to expression, visible attributes, action units, and age. These tasks support a wide range of applications, including human–computer interaction, behavioral understanding, intelligent perception, and assistive visual analysis. They have therefore been extensively studied on representative face-analysis benchmarks, such as AffectNet [1], CelebA [2], UTKFace [3], DISFA [4], BP4D [5], and DFEW [6]. Age estimation from facial images has also become an important component of human-centered vision systems [7].

Over the past decade, progress in this field has been driven mainly by stronger visual backbones, larger annotated datasets, and improved task-specific learning strategies. In facial attribute recognition, multi-label learning methods such as MOON have shown the effectiveness of task-specific attribute modeling [8]. In facial action unit analysis, attention-based and relation-aware methods, including JAA-Net and graph-based AU learning, have improved the modeling of local facial muscle movements [9,10]. In facial expression recognition, recent studies have improved robustness and dynamic modeling under unconstrained conditions [11], while benchmark construction and evaluation protocols have made experimental comparison more reliable [12]. However, many of these methods are still designed for individual tasks and are not naturally suited to unified deployment across heterogeneous face analysis settings.

To overcome the limitations of task-specific modeling, recent studies have moved toward more general and transferable face analysis frameworks. Representative methods such as FaRL, MARLIN, PrefAce, SwinFace, and Faceptor indicate that shared facial representations can support multiple downstream tasks within a common modeling pipeline [13,14,15,16,17]. More recently, multimodal large language models (MLLMs), which jointly process visual inputs and language instructions, have provided a new interface for facial understanding. Built on visual instruction-tuning frameworks such as LLaVA, LLaVA-1.5, and Video-LLaVA [18,19,20], face-oriented systems including Face-LLaVA, Emotion-LLaMA, AU-LLaVA, and VL-FAU further show that facial perception, explanation, and task prediction can be handled within a shared language-mediated framework [21,22,23,24]. Compared with conventional discriminative models, MLLM-based systems offer a unified prompt-conditioned interface and more interpretable textual interaction for different face analysis tasks.

Alongside visual evidence, practical face analysis pipelines often contain structured side information, such as estimated age, estimated gender, confidence scores, and availability indicators. Such metadata may provide useful prior information, especially when it is reliable and semantically related to the target task. A common assumption in multimodal learning is therefore that additional information can improve prediction when it complements the visual input [25,26]. However, a competing view is that auxiliary information may become harmful when it is missing, noisy, biased, or mismatched with the image content, and robust multimodal learning studies have shown that unreliable or unavailable modalities require explicit handling rather than unconditional fusion [27]. This divergence is particularly important for face analysis because age and gender are compact but semantically strong cues: they may help attribute recognition or age estimation, but they may also distract expression recognition or introduce shortcut-like behavior when their reliability is uncertain.

Despite recent progress, existing MLLM-based face analysis studies still focus mainly on visual–language alignment, instruction following, and reasoning while paying less attention to when structured metadata should be trusted and how strongly they should influence prediction. Directly injecting metadata into prompts or feature representations may improve performance under clean and well-aligned conditions, but the same strategy can increase vulnerability when metadata are degraded, incomplete, externally estimated, or semantically mismatched. The main aim of this study is therefore not only to improve metadata-assisted face analysis, but also to clarify the utility boundary of structured side information in MLLM-based facial understanding. In contrast to the common assumption that additional metadata are generally beneficial when available, we argue that metadata utility is conditional: it depends on task relevance, metadata reliability, and fusion strategy. When metadata are reliable and semantically aligned with the target task, they may provide useful auxiliary cues; however, when they are noisy, incomplete, or mismatched with the image content, they may introduce misleading priors and increase model fragility. To this end, we propose QGFace-LLaVA, a quality-aware controlled fusion framework that preserves visual dominance while regulating metadata contribution according to task context and estimated metadata reliability. In addition, we introduce counterfactual metadata reliability regularization to explicitly train the model to reduce metadata influence when side information becomes semantically inconsistent with the visual input. In this way, the proposed framework not only estimates whether metadata should be trusted under the current task, but also learns to suppress unreliable auxiliary cues under counterfactual metadata conditions.

We evaluate QGFace-LLaVA on FER2013 [12], CelebA-40 [2], and UTKFace [3], covering facial expression recognition, facial attribute recognition, and age estimation, respectively. The experiments are designed to examine metadata use under clean, progressively corrupted, missing, shuffled, and counterfactual metadata settings, thereby enabling a controlled analysis of how task relevance, metadata quality, fusion strategy, and counterfactual reliability learning affect metadata-assisted face analysis.

From a biomimetic perspective, this study is inspired by selective and reliability-weighted cue integration in biological perception. Human perception does not combine all available cues with fixed and equal weights; instead, cue contributions are adjusted according to their relative uncertainty and weakened when the cues become mutually inconsistent or are unlikely to originate from a common cause [28,29]. We adopt this principle as a computational abstraction rather than as a literal model of neural processing, treating visual evidence as the primary source and structured metadata as conditional auxiliary information whose influence should depend on reliability and consistency. The detailed correspondence between this biomimetic principle and the proposed architecture is presented in Section 3.1.

The main contributions of this work are summarized as follows:

1. We introduce a metadata utility boundary perspective for MLLM-based face analysis, showing that structured side information should be regarded as a reliability-dependent auxiliary signal rather than a uniformly beneficial input.
2. We propose QGFace-LLaVA, an MLLM-centered quality-aware controlled fusion framework in which a pretrained multimodal large language model provides the shared prompt-conditioned reasoning backbone, while structured metadata are calibrated and injected through task-aware quality estimation and gated residual correction.
3. We introduce counterfactual metadata reliability regularization to explicitly train the model to suppress metadata influence when side information becomes semantically inconsistent with the visual input.
4. We design a controlled evaluation protocol covering clean, progressively corrupted, missing, shuffled, and counterfactual metadata conditions, and further introduce the Metadata Dependence Index and metadata utility boundary analysis to examine the relationship between clean-setting utility and mismatch robustness.
5. We provide internal metadata-control analysis, component-level ablation, prompt-level sensitivity analysis, and qualitative attribution visualization to examine whether QGFace-LLaVA adaptively suppresses unreliable auxiliary cues while preserving visual evidence.

## 2. Related Work

This section reviews three research directions closely related to the present study: general face analysis, side-information fusion, and robust multimodal learning. Together, these areas provide the methodological foundation for unified face understanding and metadata-assisted prediction while revealing an unresolved problem: how compact structured metadata should be selectively controlled when their task relevance or reliability is uncertain.

### 2.1. Face Analysis

Face analysis covers a broad range of tasks, including facial expression recognition, facial attribute recognition, facial action unit detection, and age estimation. Early deep learning approaches were typically developed for individual tasks, with each task relying on a dedicated backbone, supervision strategy, and optimization objective. In facial expression recognition, in-the-wild datasets and large-scale annotation efforts, including RAF-DB and EmotioNet, enabled evaluation beyond controlled laboratory environments and under more realistic unconstrained conditions [30,31]. In age estimation, DEX and its extensions demonstrated the effectiveness of large-scale pretraining and expectation-based formulations for apparent-age and real-age prediction [32,33]. In facial action unit analysis, DSIN and OpenFace 2.0 improved structured AU modeling and practical facial-behavior analysis [34,35]. More recently, transformer-based FER methods such as TransFER and self-supervised facial pretraining approaches such as FRA have further emphasized the importance of structure-aware and transferable facial representations [36,37]. Recent AU methods, including FG-Net and MAE-Face, have also improved cross-domain generalization and representation quality [38,39].

Although these studies have achieved substantial progress on individual tasks, many remain specialized to isolated benchmarks or task-specific settings. To improve scalability and transferability, recent research has moved toward broader, more transferable, and increasingly unified face-analysis representations. ArcFace demonstrated the importance of discriminative face representation learning at scale, FairFace highlighted the role of balanced demographic data in face analytics, and FaceXFormer extended transformer-based modeling to multiple facial-analysis tasks within a unified architecture [40,41,42]. In parallel, face-oriented language-grounded systems, including FaceLLM and interactive explanation frameworks, indicate that face analysis is evolving from conventional discriminative prediction toward more interpretable and language-mediated understanding [43,44].

However, most existing studies primarily emphasize visual representation learning and task prediction while offering limited guidance on how structured metadata should be selectively used across heterogeneous face-analysis tasks. In particular, they seldom examine whether metadata relevance and reliability vary across expression recognition, attribute prediction, and age estimation. This limitation motivates the present study to investigate task-dependent and reliability-aware metadata control within a unified MLLM-based face-analysis framework.

### 2.2. Side-Information Fusion

Side-information fusion has been widely studied in multimodal learning, where visual representations are combined with auxiliary structured variables to improve prediction. Early fusion strategies commonly relied on direct concatenation or fixed-strength feature combination. Later approaches introduced learnable conditioning mechanisms to regulate the influence of auxiliary inputs on visual features. For example, FiLM performs feature-wise affine modulation according to conditioning information, whereas DAFT dynamically transforms image features using auxiliary tabular variables [45,46]. More recent image–tabular learning methods have further shown that structured information can provide complementary task-relevant cues through contrastive pretraining, dynamic fusion, and semi-supervised multimodal learning [47,48,49]. Multimodal transformer formulations also provide a broader perspective on heterogeneous modality interaction through cross-modal attention, including cases in which the participating modalities are not perfectly aligned [50]. Reviews of image and non-image data fusion similarly emphasize that multimodal benefit depends not only on the availability of additional information, but also on its effective extraction, alignment, and integration [51].

Recent credibility-aware multimodal fusion methods have begun to model the reliability of individual information sources explicitly. Credibility-Weighted Mean (CWM), for example, estimates modality credibility using probabilistic circuits and regulates the contribution of each modality accordingly [52]. This work demonstrates that auxiliary information should not be treated as uniformly trustworthy. However, CWM primarily estimates source-level credibility rather than task-conditioned metadata quality, and it does not explicitly train the model to suppress semantically mismatched structured metadata through counterfactual perturbations.

These limitations are particularly relevant to face analysis. Compact metadata such as age, gender, confidence scores, and availability indicators are low-dimensional but semantically influential. Their utility may differ substantially across tasks: age-related information may be directly relevant to age estimation, partially useful for facial attribute prediction, and less informative for facial expression recognition. Moreover, automatically estimated metadata may be noisy, missing, or inconsistent with the corresponding image. Consequently, the value of side information depends jointly on task relevance, metadata reliability, and the mechanism used to regulate its contribution.

Taken together, the representative strategies discussed above provide important foundations for auxiliary-information integration. However, none of the approaches summarized in Table 1 jointly combines explicit reliability estimation, task-aware metadata-quality modeling, controlled auxiliary influence, counterfactual mismatch training, and systematic evaluation under imperfect inputs. In Table 1, a check mark indicates that the corresponding capability is explicitly modeled or evaluated, whereas a dash indicates that it is absent or not explicitly reported.

Table 1 shows that the novelty of QGFace-LLaVA does not lie in introducing side-information fusion alone. FiLM and DAFT condition visual features on auxiliary inputs, whereas CWM additionally regulates multimodal aggregation according to source credibility. In contrast, QGFace-LLaVA estimates metadata quality under the current task context and constrains auxiliary influence through controlled residual fusion while preserving visual dominance. The full framework further introduces counterfactual metadata reliability regularization to suppress semantically inconsistent metadata during training and is evaluated under noisy, missing, shuffled, counterfactual, and naturally occurring metadata errors. This combination distinguishes QGFace-LLaVA from conventional conditioning and source-level credibility-aware fusion strategies.

### 2.3. Robust Multimodal Learning

Robust multimodal learning investigates how models behave when one or more information sources are missing, noisy, or unreliable. HeMIS established the principle that multimodal systems should remain functional when some modalities are unavailable rather than assuming complete multimodal inputs [53]. Subsequent work showed that even strong multimodal transformers can be sensitive to missing modalities [54]. To improve robustness, researchers have explored uncertainty-aware fusion and shared-specific representation learning [55,56], as well as modality imagination, prompt-based compensation, parameter-efficient adaptation, and missing-modality prediction [57,58,59,60]. More recent frameworks, including HEALNet [61] and credibility-aware probabilistic fusion [52], further support flexible multimodal integration under heterogeneous-, incomplete-, or noisy-input conditions.

Most of these approaches focus on general multimodal classification, biomedical fusion, sentiment analysis, or the recovery of missing high-dimensional modalities. The setting considered in this study is different: compact metadata such as age, gender, confidence scores, and availability indicators may remain present while becoming unreliable or semantically inconsistent with the corresponding face image. Because these variables carry explicit semantic priors, unconditional fusion can directly bias prediction. Robustness in this setting therefore requires not only missing-input handling, but also explicit decisions about when metadata should be trusted, weakened, or suppressed.

QGFace-LLaVA addresses this metadata-specific problem through task-aware quality estimation, controlled metadata fusion, and counterfactual metadata reliability regularization. It therefore extends robust multimodal learning from conventional missing-modality handling toward reliability-aware control of structured side information in MLLM-based face analysis.

## 3. Methods

To address the conditional utility of structured side information under imperfect metadata, we propose QGFace-LLaVA, a quality-aware controlled fusion framework for robust MLLM-based face analysis. Instead of directly concatenating metadata with visual representations or injecting them with fixed strength, QGFace-LLaVA estimates metadata reliability under the current task context and regulates metadata influence while preserving the dominant role of visual evidence. Accordingly, structured side information is treated as a conditional auxiliary signal rather than a uniformly beneficial input, and its contribution is determined jointly by metadata reliability, task relevance, and fusion control.

**Figure 1:**
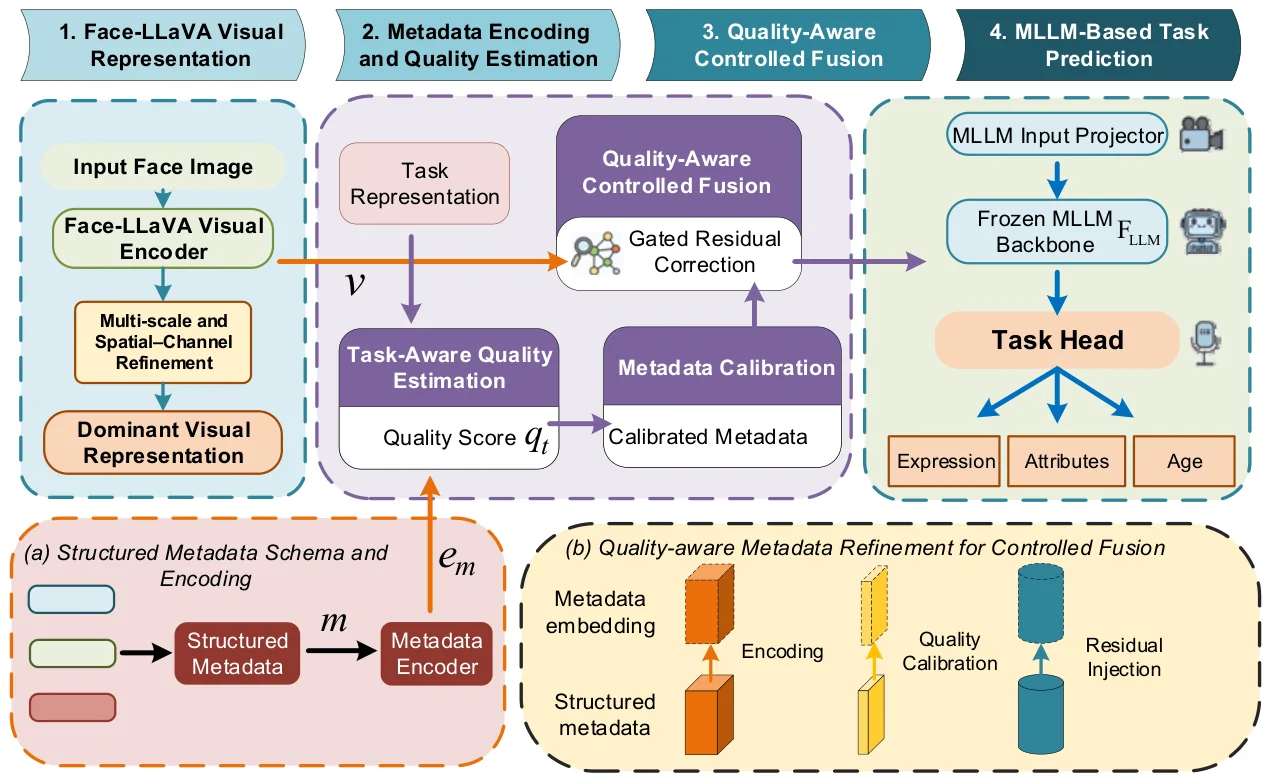
Overall architecture of QGFace-LLaVA. The upper panel presents a four-stage inference pipeline comprising visual representation extraction and refinement using a frozen Face-LLaVA visual encoder, structured metadata encoding and task-aware quality estimation, quality-aware controlled fusion, and MLLM-based task prediction using a frozen MLLM backbone. (**a**) Structured metadata schema and encoding. (**b**) Quality-aware metadata calibration and gated residual correction.

As illustrated in Figure 1, the inference pipeline consists of four stages: dominant visual representation construction, structured metadata encoding with task-aware reliability estimation, quality-aware controlled fusion, and prompt-conditioned task prediction through an MLLM backbone. During training, counterfactual metadata reliability regularization is further introduced to reduce over-dependence on semantically inconsistent metadata.

### 3.1. Overall Framework

Given an input face image x, structured metadata m, and a prompt-derived task representation $p_{t}$, QGFace-LLaVA regulates whether the metadata should be trusted and how strongly they should influence prediction under task t. The key principle is to preserve the visual representation as the dominant evidence while using structured metadata as a task-aware and reliability-controlled auxiliary signal.

This design follows three biomimetic correspondences. First, the dominant visual pathway reflects cue dominance, in which the primary perceptual source remains responsible for the main task prediction. Second, the task-aware quality score implements reliability-dependent cue weighting by estimating whether the structured metadata are sufficiently reliable and task-relevant for the current sample. Third, quality calibration and gated residual correction implement adaptive cue integration by allowing auxiliary metadata to modify the visual representation only when their estimated reliability and semantic compatibility are sufficiently high. CMRR extends this correspondence to inconsistent-cue conditions by explicitly reducing metadata influence when the auxiliary information no longer agrees with the visual evidence.

The inference pipeline consists of four stages: dominant visual representation construction, structured metadata encoding with task-aware reliability estimation, quality-aware controlled fusion, and prompt-conditioned prediction in a shared MLLM task space. These stages are formulated as follows:

(1) $$v=D\left(E_{v}\left(x\right)\right)$$

(2) $$\left(e_{m},q_{t},r_{m}\right)=E_{m}\left(m,v,p_{t}\right)$$

(3) $$z_{t}=G\left(v,r_{m},p_{t}\right)$$

(4) $${\hat{y}}_{t}=H_{t}\left(F_{LLM}\left(P_{f}\left(z_{t}\right),p_{t}\right)\right)$$

Here, $E_{v}(\cdot )$ denotes the visual encoder, and D(⋅) denotes dominant visual representation construction. The resulting representation, v, serves as the visual anchor for subsequent metadata regulation. $E_{m}(\cdot )$ denotes the metadata pathway, which takes m, v, and $p_{t}$ as inputs and produces the latent metadata embedding, $e_{m}$, the task-aware metadata reliability score, $q_{t}$, and the fusion-space metadata representation,${\;r}_{m}$, G(⋅) denotes the quality-aware controlled fusion module, which generates the fused representation, $z_{t}$, under task t. $P_{f}(\cdot )$, $F_{LLM}(\cdot )$, and $H_{t}(\cdot )$ denote the fusion projector, the pretrained MLLM backbone, and the task-specific prediction head, respectively. The final output, ${\hat{y}}_{t}$, denotes the task-specific prediction.

The detailed formulations of Equations (1)–(4) are presented in Section 3.2, Section 3.3, Section 3.4, Section 3.5 and Section 3.6. Specifically, Section 3.2 describes dominant visual representation construction, Section 3.3 introduces structured metadata encoding and task-aware reliability estimation, Section 3.4 presents quality-aware controlled fusion, Section 3.5 describes the training-only counterfactual metadata reliability regularization strategy, and Section 3.6 explains MLLM-based task prediction and optimization.

### 3.2. Dominant Visual Representation Construction

The visual branch constructs a dominant visual representation that serves as a stable visual anchor for subsequent metadata regulation [16]. Given an input face image x, the visual encoder first extracts an intermediate feature map:

(5) $$F=E_{v}\left(x\right)$$

where $E_{v}(\cdot )$ denotes the visual encoder and F denotes the intermediate visual feature map. A compact global visual descriptor is then obtained by global average pooling:

(6) $$v_{g}=GAP\left(F\right)$$

where $v_{g}$ denotes the global visual representation and GAP(⋅) denotes global average pooling.

**Figure 2:**
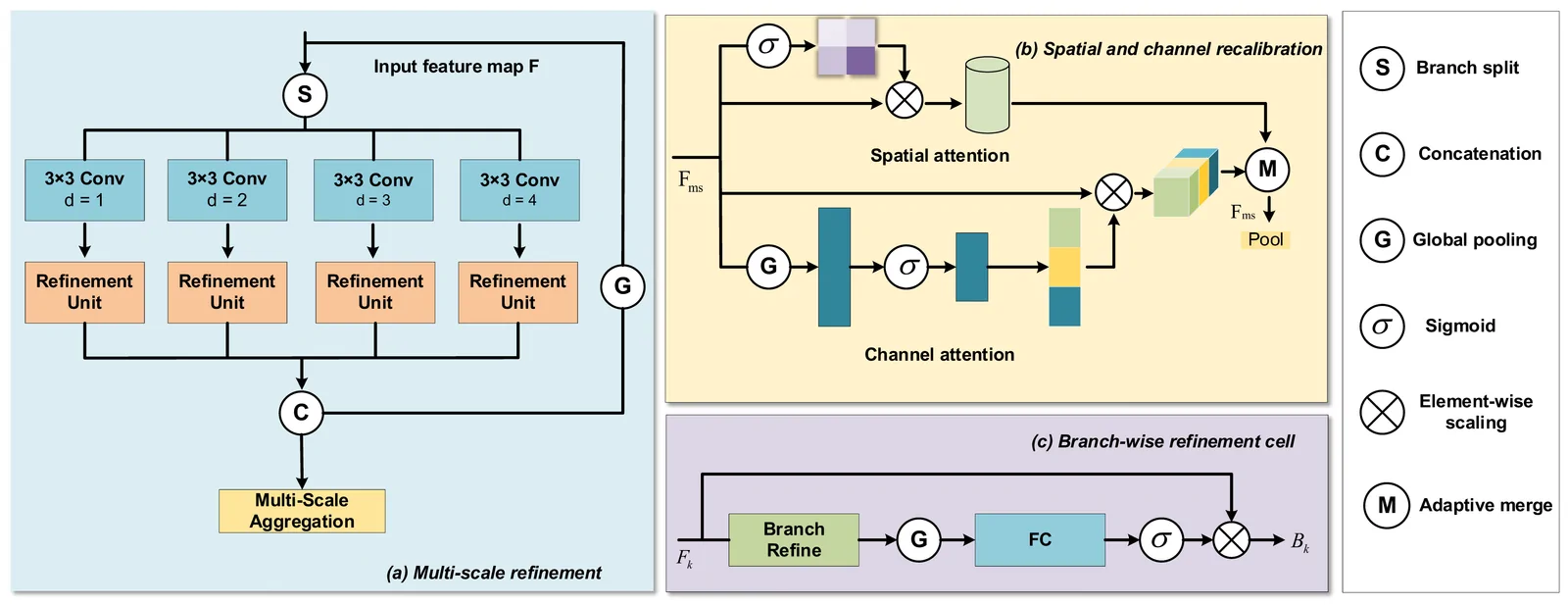
Visual branch for dominant visual representation construction. (**a**) Parallel dilated convolution branches perform multi-scale refinement and feature aggregation. (**b**) Spatial and channel recalibration emphasizes informative responses before global pooling. (**c**) A lightweight refinement cell recalibrates each branch before aggregation.

To capture facial cues at different receptive fields, a multi-scale refinement branch is introduced, as illustrated in Figure 2a. The intermediate feature map is processed by K parallel dilated branches. For the k-th branch, the refined response is defined as follows:

(7) $$B_{k}=R_{k}\left({Conv}_{d_{k}}\left(F\right)\right),k=1,2,\ldots ,K$$

where ${Conv}_{d_{k}}(\cdot )$ denotes convolution with dilation factor $d_{k}$, $R_{k}(\cdot )$ denotes the branch-wise refinement cell, and $B_{k}$ denotes the refined response of the k-th branch. The branch responses are then aggregated into a unified multi-scale feature map:

(8) $$F_{ms}=A_{b}\left(Concat\left(B_{1},B_{2},\ldots ,B_{K}\right)\right)$$

where Concat(⋅) denotes feature concatenation and $A_{b}(\cdot )$ denotes branch aggregation.

To emphasize informative visual responses, the aggregated multi-scale feature map is recalibrated along spatial and channel dimensions, as illustrated in Figure 2b:

(9) $${\hat{F}}_{ms}=M\left(S\left(F_{ms}\right),C\left(F_{ms}\right)\right)$$

where S(⋅) and C(⋅) denote spatial recalibration and channel recalibration, respectively, M(⋅) denotes adaptive merging, and ${\hat{F}}_{ms}$ denotes the recalibrated multi-scale feature map. Finally, the recalibrated multi-scale feature is compressed and combined with the global descriptor to form the dominant visual representation:

(10) $$v_{ms}=GAP\left({\hat{F}}_{ms}\right),v=\alpha v_{g}+\beta v_{ms}$$

where $v_{ms}$ denotes the pooled multi-scale representation, and α and β are learnable coefficients that balance the global and multi-scale components. In this way, v preserves both global semantic stability and refined local structural sensitivity, providing a reliable visual anchor for subsequent quality-aware metadata regulation.

Overall, the visual branch provides a shared and stable visual anchor for all compared variants. The main methodological focus of QGFace-LLaVA is placed on reliability-aware control of structured metadata rather than on introducing a new visual backbone.

### 3.3. Structured Metadata Encoding and Task-Aware Quality Estimation

To encode structured metadata in a unified and task-compatible form, the metadata branch represents each sample using five channels: normalized age, gender, an age-confidence indicator, gender confidence, and a metadata availability indicator. Let a denote raw age metadata, g denote gender metadata, $c_{a}$ and $c_{g}$ denote the confidence scores of age and gender, respectively, and s∈{0,1} denote the metadata availability indicator. The age-confidence and gender-confidence channels are normalized to the range [0, 1] and are used as relative reliability cues rather than calibrated probabilities of prediction correctness. The normalized age value is defined as follows:

(11) $$a_{n}=\frac{a-\mu_{a}}{\sigma_{a}}$$

where $a_{n}$ denotes the normalized age value, and $\mu_{a}$ and $\sigma_{a}$ denote the mean and standard deviation of age computed from the training split, respectively. The structured metadata vector is then written as follows:

(12) $$m={\left[a_{n},g,c_{a},c_{g},s\right]}^{T}$$

where m denotes the five-channel metadata vector. This representation integrates informative metadata values, confidence cues, and availability information into a unified schema, allowing the model to distinguish between reliable, uncertain, and unavailable side information.

The structured metadata vector is projected into a latent embedding space by:

(13) $$e_{m}=\phi \left(W_{m}m+b_{m}\right)$$

where $W_{m}$ and $b_{m}$ are learnable parameters, ϕ(⋅) denotes a nonlinear activation function, and $e_{m}$ denotes the latent metadata embedding. This step transforms low-dimensional structured metadata into a task-compatible latent representation for reliability evaluation.

To determine whether the metadata should be trusted under the current task, a task-aware metadata reliability score is estimated by jointly considering the metadata embedding, the task representation, and the dominant visual representation:

(14) $$q_{t}=\sigma \left(W_{q}^{T}\left[e_{m};p_{t};P_{v}\left(v\right)\right]+b_{q}\right)$$

where $p_{t}$ denotes the representation of task t, $P_{v}(\cdot )$ denotes a lightweight projection that maps the dominant visual representation into the metadata-quality estimation space, $\left[\;;\;\right]$ denotes vector concatenation, $W_{q}$ and $b_{q}$ are learnable parameters, and $q_{t}\in [0,1]$ denotes the task-aware metadata reliability score. A larger $q_{t}$ indicates that the metadata are more likely to provide useful auxiliary information under the current task.

The metadata embedding is then calibrated according to the estimated reliability score:

(15) $${\tilde{e}}_{m}=q_{t}e_{m}$$

where ${\tilde{e}}_{m}$ denotes the quality-calibrated metadata embedding. Since $q_{t}$ is a scalar reliability score, this operation reduces the effective contribution of metadata when their estimated reliability is low. Finally, the calibrated metadata embedding is projected into the fusion space:

(16) $$r_{m}=P_{m}\left({\tilde{e}}_{m}\right)=W_{r}{\tilde{e}}_{m}+b_{r}$$

where $P_{m}(\cdot )$ denotes the metadata projection module, $W_{r}$ and $b_{r}$ are learnable parameters, and $r_{m}$ denotes the fusion-space metadata representation used in the subsequent controlled fusion stage. The representation $r_{m}$ is projected to be compatible with the visual representation v, enabling controlled residual fusion in Section 3.4.

Together, Equations (11)–(16) define the complete metadata pathway, including schema construction, latent encoding, task-aware reliability estimation, quality-guided calibration, and fusion-space projection. This design ensures that structured metadata are not treated as uniformly trustworthy cues, but are first evaluated under the current task context before being injected into the dominant visual representation.

### 3.4. Quality-Aware Controlled Fusion

The core of QGFace-LLaVA is to inject structured metadata into the dominant visual representation in a controlled manner. Instead of directly concatenating metadata with visual features or adding metadata with fixed strength, the proposed method treats metadata as a task-conditioned residual correction [45,48]. In this formulation, the visual representation remains the primary evidence, while metadata are allowed to influence prediction only through quality-calibrated and gate-controlled residual injection.

Given the dominant visual representation v, the fusion-space metadata representation $r_{m}$, and the task representation $p_{t}$, the dimension-wise fusion gate is defined as follows:

(17) $$g_{t}=\sigma \left(W_{g}\left[v;r_{m};p_{t}\right]+b_{g}\right)$$

where $W_{g}$ and $b_{g}$ are learnable parameters, $\sigma \left(\cdot \right)$ denotes the sigmoid function, $\left[\;;\;\right]$ denotes vector concatenation, and $g_{t}\in [0,1{]}^{d}$ is a dimension-wise fusion gate that controls metadata injection strength over the fusion space. The task-conditioned metadata residual correction is then computed as follows:

(18) $$\Delta_{t}=g_{t}⨀r_{m}$$

where ⊙ denotes element-wise multiplication. This residual term allows metadata to modify the visual representation only through the learned gate. The final fused representation is formulated as follows:

(19) $$z_{t}=v{+\Delta }_{t}$$

where $z_{t}$ denotes the fused representation under task t. Equations (17)–(19) constitute the core controlled-fusion mechanism. The quality calibration in Equation (15) reduces the contribution of unreliable metadata, while the fusion gate in Equation (17) further controls how strongly the calibrated metadata can modify the visual representation. Therefore, structured metadata are not treated as a parallel dominant modality, but as a reliability-aware auxiliary correction constrained by both task context and estimated metadata quality.

The quality score can be interpreted as a bounded surrogate for the task-conditioned reliability of the metadata. The estimated score $q_{t}$ does not represent an externally provided reliability label; instead, it is learned as a task-conditioned approximation of metadata usefulness from the metadata representation, visual evidence, task representation, and the reliability-oriented training objectives.

The quality score and fusion gate provide two complementary mechanisms for controlling metadata influence. Quality calibration attenuates the metadata representation before fusion, whereas the dimension-wise fusion gate regulates the strength of residual injection. Their effectiveness is evaluated empirically through the progressive-corruption, internal metadata-control, and component-level ablation experiments.

**Figure 3:**
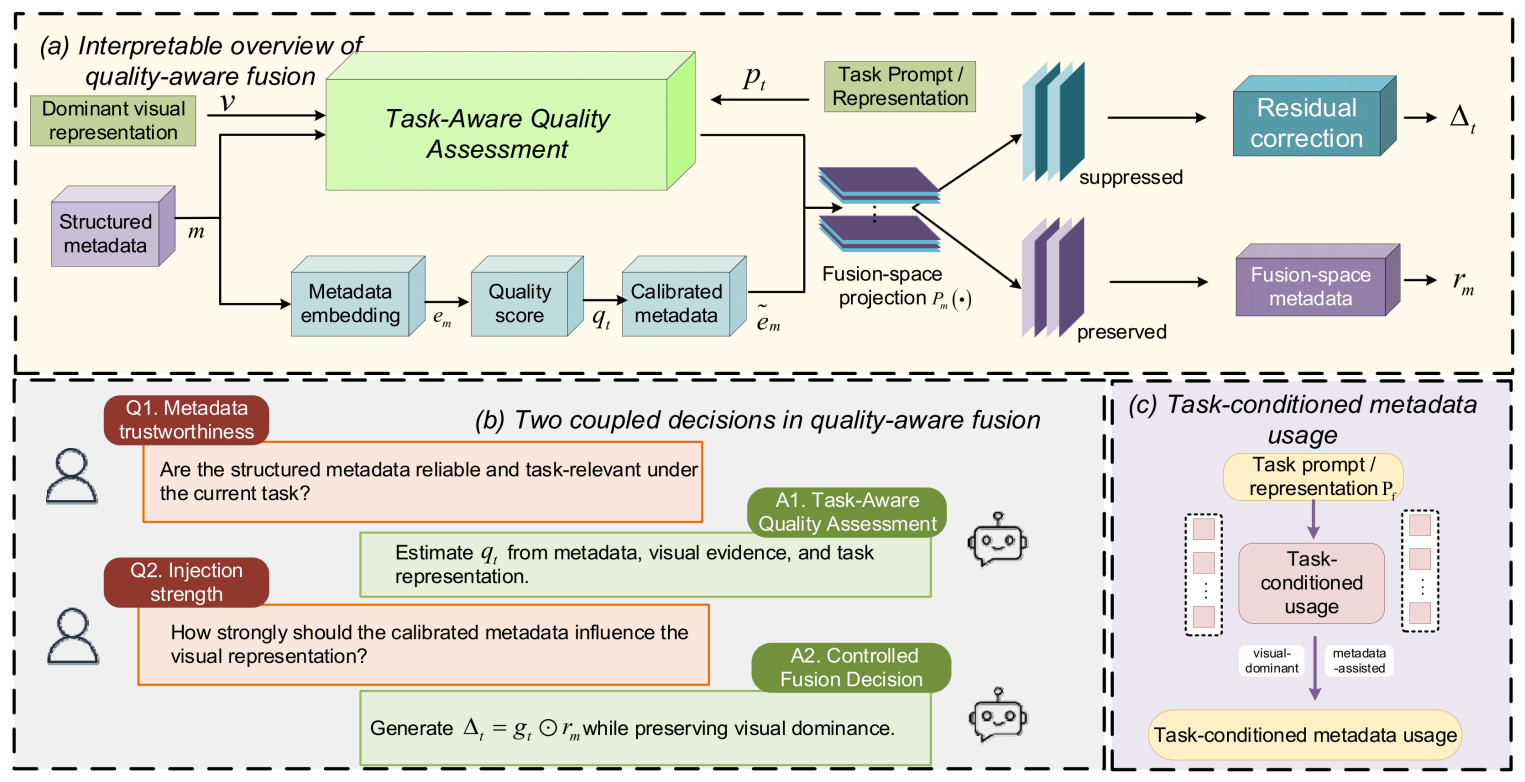
Interpretable view of quality-aware controlled fusion. (**a**) The fusion module evaluates structured metadata under the current task context and converts them into a calibrated representation for controlled residual correction. (**b**) The fusion process is interpreted as two coupled decisions: estimating metadata trustworthiness and determining metadata injection strength. (**c**) Metadata usage is task-conditioned, allowing the model to shift between visual-dominant and metadata-assisted behavior across tasks.

For interpretability, Figure 3 illustrates the fusion process from a decision-oriented perspective. It shows how metadata are evaluated, calibrated, and injected as a controlled residual correction, and how the model adaptively shifts between visual-dominant and metadata-assisted behavior across tasks. These views are explanatory and do not introduce additional independently optimized modules.

### 3.5. Counterfactual Metadata Reliability Regularization

To reduce over-dependence on semantically inconsistent metadata, we introduce a counterfactual metadata reliability regularization strategy during training. The motivation is that metadata may remain available but become mismatched with the input image. In this case, the model should preserve prediction stability and suppress the influence of unreliable side information.

Given a training sample $\left(x_{i}m_{i}y_{i}\right)$, where $x_{i}$ is the input face image, $m_{i}$ is the structured metadata, and $y_{i}$ is the task label, a counterfactual metadata vector is constructed by replacing $m_{i}$ with metadata from another sample:

(20) $$m_{i}^{cf}=m_{\pi \left(i\right)},\pi \left(i\right)\neq i$$

The image and label remain unchanged, while metadata from another sample are reassigned to disrupt the original image–metadata correspondence. The reassigned metadata are therefore treated as potentially mismatched rather than guaranteed to be semantically inconsistent with the input image. The model is then evaluated under both clean and counterfactual metadata:

(21) $${\hat{y}}_{i}^{clean}=f_{t}\left(x_{i},m_{i},p_{t}\right)$$

(22) $${\hat{y}}_{i}^{cf}=f_{t}\left(x_{i},m_{i}^{cf},p_{t}\right)$$

where $f_{t}(\cdot )$ denotes the task-conditioned prediction function. The corresponding metadata reliability scores and fusion gates are denoted as $q_{i}^{clean}$, $q_{i}^{cf}$, $g_{i}^{clean}$, and $g_{i}^{cf}$.

Since the visual input and task label are unchanged, the prediction under counterfactual metadata should remain correct and stable. Therefore, a counterfactual task loss is defined as follows:

(23) $$L_{cf-task}=l_{t}\left({\hat{y}}_{i}^{cf},y_{i}\right)$$

where $l_{t}(\cdot )$ denotes the task-specific loss. To further constrain prediction consistency between clean and counterfactual metadata, we define:

(24) $$L_{cons}=\left\{\begin{array}{c} D_{KL}\left(p_{i}^{clean}∥p_{i}^{cf}\right)\;\;\;\;\;\;\;\;\;\;\;\;\;\\ \frac{1}{C}{\left\|\sigma \left({\hat{y}}_{i}^{clean}\right)-\sigma \left({\hat{y}}_{i}^{cf}\right)\right\|}_{1}\\ \left|{\hat{y}}_{i}^{clean}-{\hat{y}}_{i}^{cf}\right|\;\;\;\;\;\;\;\;\;\;\;\;\;\;\;\;\;\;\;\;\; \end{array}\right.$$

In Equation (24), the first, second, and third rows are used for single-label classification, multi-label classification, and regression tasks, respectively. For single-label classification, $p_{i}^{clean}$ and $p_{i}^{cf}$ are obtained by applying softmax to the corresponding clean and counterfactual predictions. C denotes the number of attributes in multi-label classification, and σ(⋅) denotes the sigmoid function.

To explicitly suppress metadata reliance under counterfactual metadata, we constrain the counterfactual quality score and gate magnitude to be lower than their clean-metadata counterparts:

(25) $$L_{q}=\max\left(0,q_{i}^{cf}-q_{i}^{clean}+\delta_{q}\right)$$

(26) $${\bar{g}}_{i}=\frac{1}{d}{\left\|g_{i}\right\|}_{1},\;\;L_{g}=\max\left(0,{\bar{g}}_{i}^{cf}-{\bar{g}}_{i}^{clean}+\delta_{g}\right)$$

where ${\bar{g}}_{i}$ denotes the average gate magnitude, d is the fusion dimension, and $\delta_{q}$ and $\delta_{g}$ are margin hyperparameters. The overall counterfactual metadata reliability regularization loss is defined as follows:

(27) $$L_{CMRR}=\lambda_{cf}L_{cf-task}+\lambda_{cons}L_{cons}+\lambda_{sup}\left(L_{q}+L_{g}\right)$$

where $\lambda_{cf}$, $\lambda_{cons}$, and $\lambda_{sup}$ control the relative strengths of counterfactual task supervision, prediction consistency, and metadata suppression, respectively. By incorporating counterfactual metadata during training, the model learns to maintain prediction stability while reducing metadata influence when side information becomes semantically mismatched with the visual input.

### 3.6. MLLM-Based Task Prediction and Optimization

After quality-aware controlled fusion, the fused representation is projected into the MLLM-aligned task space, where the pretrained MLLM serves as the shared prompt-conditioned reasoning backbone for heterogeneous face analysis. Rather than treating the MLLM as a passive classifier, QGFace-LLaVA uses it as a unified visual–language interface that links task prompts, fused visual–metadata representations, and downstream task-specific predictions. In this way, facial expression recognition, facial attribute recognition, and age estimation can be formulated within a common MLLM-based prediction framework, while the proposed metadata-control modules determine how reliable auxiliary information should enter this reasoning process:

(28) $$u_{t}=P_{f}\left(z_{t}\right),h_{t}=F_{LLM}\left(u_{t},p_{t}\right)$$

where $P_{f}(\cdot )$ denotes the fusion projector, $F_{LLM}(\cdot )$ denotes the pretrained MLLM backbone, $u_{t}$ denotes the projected representation, and $h_{t}$ denotes the task-conditioned hidden representation. Different task prompts guide the same fused representation toward different downstream objectives, including facial expression recognition, facial attribute recognition, and age estimation.

The final task output is obtained through a task-specific prediction head. For CelebA-40, the task-specific prediction head produces 40 independent scalar logits, with one logit corresponding to each facial attribute:

(29) $${\hat{y}}_{t}=H_{t}\left(h_{t}\right)$$

where $H_{t}(\cdot )$ denotes the task-specific prediction head for task t, and ${\hat{y}}_{t}$ denotes the final prediction. The task loss is defined according to the downstream task:

(30) $$L_{task}=\left\{\begin{array}{c} L_{CE}\left({\hat{y}}_{t},y_{t}\right),\;\;\;\;\;\;\;t\in T_{cls}\\ L_{BCE}\left({\hat{y}}_{t},y_{t}\right),\;\;\;\;\;t\in T_{ml}\\ L_{MAE}\left({\hat{y}}_{t},y_{t}\right)\;,\;\;\;t\in T_{reg} \end{array}\right.$$

Here, $y_{t}$ denotes the ground-truth target. $T_{cls}$, $T_{ml}$, and $T_{reg}$ denote facial expression classification, multi-label facial attribute recognition, and age regression, respectively; $L_{CE}$, $L_{BCE}$, and $L_{MAE}$ denote cross-entropy, binary cross-entropy, and mean absolute error losses, respectively. To prevent excessive metadata reliance and preserve visual dominance, two regularization terms are introduced. The gate regularization term discourages overly strong metadata injection:

(31) $$L_{gate}=\frac{1}{d}{∥g_{t}∥}_{1}$$

where d denotes the fusion dimension. The visual-preservation term constrains the fused representation not to deviate excessively from the dominant visual representation:

(32) $$L_{vis}={∥z_{t}-v∥}_{2}^{2}$$

The overall optimization objective is defined as follows:

(33) $$L=L_{task}+{\lambda_{cmrr}L}_{CMRR}+\lambda_{g}L_{gate}+\lambda_{v}L_{vis}$$

Here, $\lambda_{cmrr}$, $\lambda_{g}$, and $\lambda_{v}$ are weighting coefficients for counterfactual metadata reliability regularization, gate regularization, and visual-preservation regularization, respectively. For the standard QGFace-LLaVA variant, $\lambda_{cmrr}=0$; for QGFace-LLaVA with counterfactual metadata reliability regularization, $\lambda_{cmrr}>0$.

Overall, Equation (33) reflects the central optimization principle of QGFace-LLaVA: structured metadata should not dominate prediction, but should serve as a quality-aware auxiliary correction to the visual branch. The framework jointly optimizes task performance, counterfactual metadata robustness, conservative metadata usage, and preservation of visual dominance.

## 4. Results

This section evaluates the proposed QGFace-LLaVA architecture and the additional robustness obtained through CMRR-enhanced training under Clean, degraded, missing, semantically mismatched, and naturally erroneous metadata conditions. The experiments address three questions: (1) whether the utility of structured metadata varies with its expected relevance to the target task under the Clean condition; (2) whether the quality-aware controlled-fusion architecture improves robustness relative to simpler metadata-usage strategies, and whether CMRR provides further robustness gains during training; and (3) whether clean-setting gain and robustness under metadata mismatch are monotonically related. Together, these analyses clarify when structured side information is beneficial, unreliable, or potentially harmful in MLLM-based face analysis.

### 4.1. Datasets and Data Partitions

Experiments were conducted on FER2013 [12], CelebA-40 [2], and UTKFace [3], covering single-label facial-expression classification, multi-label facial-attribute recognition, and age regression, respectively. To analyze the effect of task relevance, the three datasets were treated as qualitative low-, moderate-, and high-alignment settings according to the expected relevance of the age- and gender-related metadata to each prediction objective. On FER2013, demographic metadata are only indirectly related to facial-expression recognition. On CelebA-40, they are relevant to a subset of the 40 facial attributes but are not uniformly informative across all attributes. On UTKFace, estimated age and gender are more directly related to the age-regression objective. These qualitative alignment levels provide a cross-task spectrum for examining whether the observed utility of structured metadata is broadly consistent with its expected relevance to each task. Because the datasets also differ in task formulation, scale, label structure, and evaluation metric, the resulting comparisons are interpreted as qualitative cross-task evidence rather than as a strictly controlled isolation of task relevance.

For FER2013, we followed the original benchmark partition, using the Training subset for model fitting, PublicTest for validation, and PrivateTest exclusively for final evaluation. For CelebA-40, the officially released training, validation, and test partitions were used without additional subsampling. Because UTKFace does not provide a single standardized official partition, all 24,106 valid images were divided using a fixed age-group- and gender-stratified 72%/8%/20% split with seed 42, resulting in 17,355 training images, 1929 validation images, and 4822 test images. The partitions were fixed before model training and reused across the core QGFace-LLaVA architecture, the CMRR-enhanced configuration, all controlled baselines, external comparison methods, training seeds, metadata conditions, and component-level ablations.

Table 2 summarizes the task definitions, qualitative metadata-alignment settings, fixed dataset partitions, and primary evaluation metrics. The metadata-degradation, missingness, and semantic-mismatch conditions used in the robustness experiments are formally defined in Section 4.2.3.

The metadata-alignment levels are qualitative experimental designations based on the expected relevance of the estimated age and gender metadata to each target task. They are not annotations provided by the original datasets and are used only to support comparative cross-task interpretation.

### 4.2. Compared Methods and Evaluation Protocol

All controlled variants were evaluated using the fixed dataset partitions and evaluation samples defined in Section 4.1. They shared the same frozen Face-LLaVA backbone and task-specific prediction interface, differing only in how structured metadata were encoded and regulated. External baselines were reproduced on the same partitions using their corresponding model-specific training protocols. The following subsections describe the compared methods, metadata construction, perturbation protocol, implementation settings, and efficiency evaluation.

#### 4.2.1. Controlled Variants and External Baselines

The controlled comparison included four inference architectures and one CMRR-enhanced training configuration. Base used only the visual representation produced by the frozen Face-LLaVA backbone and received no structured metadata. Naive Fusion directly concatenated the encoded five-channel metadata representation with the projected visual feature, without estimating metadata reliability. Gate Only was implemented as an independently constructed gated-fusion baseline. It uses a learnable gate to regulate metadata injection but does not include task-aware quality estimation, quality-guided metadata calibration, or CMRR-enhanced training. Its purpose is to evaluate whether adaptive gating alone is sufficient for metadata integration. This baseline is distinct from the Full w/o quality-aware regulation variant introduced in Section 4.4.4. The latter is derived directly from the full QGFace-LLaVA configuration by removing the quality-aware regulation block while retaining the remaining model-specific fusion pathway and applicable training constraints. QGFace-LLaVA denotes the proposed quality-aware controlled-fusion architecture, combining task-aware quality estimation, reliability-guided metadata calibration, and gated residual correction while preserving visual dominance. QGFace-LLaVA + CMRR uses the same inference architecture but additionally applies counterfactual metadata reliability regularization during training.

External baselines were selected to provide recent, strong, and reproducible comparisons for each face-analysis task. The selection considered task relevance, implementation availability, and coverage of recent unified or established task-specific methods. Accordingly, ViT for Small-Size Datasets [62] and GLRLDLW [63] were evaluated on FER2013, Faceptor-Full [17] and FaceXFormer [41] on CelebA-40, and AnyFace++ [64] and CORAL-CNN [65] on UTKFace. Model-specific preprocessing, objectives, and optimization settings followed the corresponding official implementations. When adaptation was necessary, only the dataset interface and task-specific output layer were modified. All external baselines were trained using seeds 42, 2024, and 3407, and the results are reported as mean ± standard deviation.

#### 4.2.2. Metadata Generation and Leakage Prevention

All metadata-assisted variants used the same five-channel structured metadata vector: $m_{i}=\left[{\tilde{a}}_{i}\;{\hat{g}}_{i}\;c_{i}^{a}\;c_{i}^{g}\;r_{i}\right]$, where ${\tilde{a}}_{i}$ is the normalized estimated age, ${\hat{g}}_{i}$ is the estimated gender, $c_{i}^{a}$ and $c_{i}^{g}$ are the corresponding confidence scores, and $r_{i}$ indicates metadata availability. Age and gender estimates were obtained from each facial image using a frozen MiVOLO estimator [66]. Because MiVOLO does not provide a native calibrated confidence score for its continuous age estimate, the age-confidence channel was implemented as a condition-dependent reliability indicator. It was set to 1 for available unperturbed metadata; under the test-time perturbation protocol, it was multiplied by 0.50 for samples selected under the Noisy conditions and set to 0 for samples selected under the Missing condition. It was therefore not interpreted as a sample-specific probability of age-estimation correctness. The gender-confidence channel retained the estimator-provided confidence and was used as an auxiliary reliability cue. Both confidence channels were used only for downstream metadata-quality estimation and fusion control and were not used as task labels or evaluation targets. The MiVOLO checkpoint and preprocessing pipeline were fixed before downstream training, and all metadata were generated once and cached separately for the training, validation, and test partitions.

MiVOLO received only the facial image and was not provided with task labels or ground-truth demographic attributes. On UTKFace, filename-derived age was used as the regression target, while filename-derived age and gender were used only to construct the stratified partition described in Section 4.1. Neither ground-truth age nor ground-truth gender was included in the metadata vector. Test labels were not used for metadata generation, normalization, checkpoint selection, or hyperparameter tuning.

For each dataset, the estimated-age channel was standardized using statistics calculated exclusively from the MiVOLO-estimated ages in the training partition: ${\tilde{a}}_{i}=\frac{{\hat{a}}_{i}-\mu_{train}}{\sigma_{train}},$ where ${\hat{a}}_{i}\;$ denotes the raw MiVOLO age estimate, and $\mu_{train}$ and $\sigma_{train}\;$ denote the corresponding training-partition mean and standard deviation. The same statistics were applied unchanged to the validation and test partitions.

Estimated gender was encoded as 0 for female and 1 for male. The age- and gender-confidence scores were retained on the $\left[0,\;1\right]\;$ scale, and the clean-condition availability indicator was set to 1 whenever valid metadata were available. The same encoding and normalization procedure was used across all metadata-assisted variants. Counterfactual metadata for CMRR were constructed only within the current training mini-batch and were never generated from validation or test samples.

Structured metadata were used during both downstream training and inference for all metadata-assisted variants. However, metadata were not strictly required at inference. When metadata were unavailable, the estimated-age, estimated-gender, age-confidence, and gender-confidence channels were set to zero, while the availability indicator was set to 0. This representation explicitly informed the model that the auxiliary information was unavailable and allowed prediction to rely primarily on visual evidence. In contrast, the Base variant used only visual information during both training and inference.

#### 4.2.3. Evaluation Protocol and Metadata Perturbation Settings

All controlled variants were trained and selected using the original, unperturbed metadata available in the training and validation partitions. For each method and training seed, the best validation checkpoint was evaluated on the fixed test partition under Clean, Noisy20, Noisy40, Noisy60, Noisy80, Missing60, and Shuffled metadata conditions. Here, Clean denotes the original MiVOLO-estimated metadata without any additional artificial perturbation; it does not imply that the metadata are error-free, because naturally occurring MiVOLO estimation errors may still be present. The same checkpoint was used across all test-time conditions without condition-specific fine-tuning, reselection, or hyperparameter adjustment. Base received only visual information. The CMRR-enhanced configuration used the same QGFace-LLaVA inference architecture and test-time inputs as the core model, differing only in its training objective.

Metadata perturbations were generated after the dataset partitions had been fixed, without altering the original image–label correspondence. For each dataset, test samples were ordered once using perturbation seed 2026. Noisy20, Noisy40, Noisy60, and Noisy80 corrupted the first 20%, 40%, 60%, and 80% of this fixed ordering, respectively, resulting in nested subsets: Noisy20⊂Noisy40⊂Noisy60⊂Noisy80. A fixed Gaussian age perturbation was generated for each test sample and reused across corruption levels and methods.

For samples selected under a Noisy condition, Gaussian noise was added to the normalized estimated-age channel: ${\tilde{a}}_{i}^{noisy}=\mathrm{clip}\left({\tilde{a}}_{i}+\varepsilon_{i}\;z_{\min,train}\;z_{\max,train}\right),\varepsilon_{i}∼N\left(00.50^{2}\right)$, where $z_{\min,train}$ and $z_{\max,train}$ denote the minimum and maximum normalized estimated-age values in the training partition. For the same samples, estimated gender was flipped, both confidence scores were multiplied by 0.50, and the availability indicator remained 1. The Noisy conditions therefore represented degraded but still available metadata.

Missing60 used the same fixed 60% subset as Noisy60. For selected samples, the estimated-age, estimated-gender, and two confidence channels were set to zero, while the availability indicator was set to 0. The remaining samples retained clean metadata.

Under Shuffled metadata, complete five-channel vectors were reassigned across the fixed test partition using a derangement generated with seed 2026, ensuring that no sample retained its original metadata vector. All channels were moved together, preserving their marginal distributions and internal consistency while breaking image–metadata correspondence. Metadata were not exchanged across the training, validation, and test partitions.

FER2013 was evaluated using classification accuracy, CelebA-40 using mean attribute accuracy (mAcc), and UTKFace using mean absolute error (MAE, in years). Let $S_{clean}$ and $S_{P}$ denote the performance under clean metadata and an imperfect-metadata condition P, respectively. To ensure that larger values consistently represent worse performance, the direction-aware performance degradation was defined as follows: for accuracy and mAcc: $\Delta_{P}=S_{clean}-S_{P}$; for MAE: $\Delta_{P}=S_{P}-S_{clean}$. Therefore, a positive $\Delta_{P}$ consistently indicates performance degradation. Sensitivity to semantic mismatch was quantified using the Metadata Dependence Index (MDI): $MDI=|S_{clean}-S_{shuffled}|.$ MDI is expressed in percentage points for FER2013 and CelebA-40 and in years for UTKFace. Smaller values indicate lower mismatch sensitivity, and values are interpreted only within the same dataset.

#### 4.2.4. Implementation Details and Computational Efficiency

All controlled variants were implemented in Python 3.10.20 using PyTorch 2.8.0 and CUDA 12.8 under Ubuntu 22.04. Experiments were conducted on an NVIDIA GeForce RTX 5090 D GPU with 32 GB of memory, 25 Intel Xeon Platinum 8470Q vCPU cores, and 90 GB of system memory. Input images were resized to 336×336 pixels. The pretrained Face-LLaVA backbone was frozen throughout downstream training, while the visual projection, metadata-processing, fusion or gating, and task-specific prediction modules were optimized according to the corresponding variant.

The 4096-dimensional backbone representation was projected into a 256-dimensional fusion space. The five-channel metadata vector was encoded using a hidden dimension of 128, and the task-aware quality estimator used a hidden dimension of 64 to produce a scalar reliability score. The prediction-head output dimensions were 7, 40, and 1 for FER2013, CelebA-40, and UTKFace, respectively.

Trainable modules were optimized using AdamW with an initial learning rate of $1\times 10^{-4}$, a weight decay of $1\times 10^{-4}$, and a batch size of 16. A cosine learning-rate schedule with a warm-up ratio of 0.05 was applied for a maximum of 30 epochs. Checkpoints were selected using validation accuracy for FER2013, validation mAcc for CelebA-40, and validation MAE for UTKFace. A small preliminary hyperparameter search was conducted using only the clean training and validation partitions with seed 42. The learning rate, fusion dimension, metadata-encoder dimension, quality-estimator dimension, and regularization coefficients were varied. Candidate configurations were evaluated separately on the clean validation partition of each dataset using its corresponding task-specific metric, and the resulting validation performance was considered jointly to retain a single shared configuration across the three tasks. The task-specific validation metrics were not combined into a single numerical score. After selection, all hyperparameters were fixed for the controlled variants evaluated with seeds 42, 2024, and 3407 and under all test-time metadata conditions. The test partitions were not used for hyperparameter selection, checkpoint reselection, or condition-specific adjustment. External baselines retained their official model-specific optimization protocols.

For the CMRR-enhanced configuration, counterfactual reliability regularization was activated after the first five epochs. Following Section 3.6, the counterfactual task, prediction-consistency, and metadata-suppression loss weights were set to 1.0, 0.5, and 0.1, respectively. The quality-score and gate-magnitude margins were both set to 0.05, while the gate-regularization and visual-preservation weights were set to $1\times 10^{-3}$ and $1\times 10^{-2}$, respectively. These settings were fixed across datasets, seeds, and metadata conditions.

Unless otherwise specified, all subsequent controlled experiments used the fixed dataset partitions, checkpoint-selection procedure, training seeds, and test samples described above. The results are reported as mean ± standard deviation over checkpoints trained with seeds 42, 2024, and 3407.

Computational efficiency was evaluated by separating the shared cost of the frozen Face-LLaVA backbone from the incremental overhead of the trainable task-head and metadata-fusion modules. The CelebA-40 configuration was used because its 40-dimensional prediction head was the largest among the evaluated tasks. Trainable parameter counts and incremental MFLOPs included all method-specific projection, metadata-encoding, quality-estimation, calibration, gating, residual-fusion, and prediction-head layers. The common 7.148-billion-parameter Face-LLaVA backbone was excluded from these incremental counts.

Incremental latency was measured under FP16 inference with a batch size of 1. Gradient computation was disabled using PyTorch inference mode. Each measurement consisted of 200 warm-up forward passes followed by 2000 timed passes, with CUDA synchronization applied immediately before and after timing. The procedure was independently repeated five times, and latency is reported as mean ± standard deviation. The shared backbone, model loading, disk input/output, image preprocessing, and offline MiVOLO generation were excluded from the incremental latency benchmark.

As shown in Table 3, the trainable task-head and metadata-fusion modules of QGFace-LLaVA contain 1.231 million parameters, require 2.457 MFLOPs per sample, and incur 0.205 ± 0.010 ms of incremental module-level inference latency. Relative to Base, the additional overhead is 0.171 million parameters, 0.339 MFLOPs, and 0.155 ms. Because the frozen Face-LLaVA backbone is shared across all controlled variants, these measurements isolate the method-specific computational cost. CMRR modifies only the training objective and therefore adds no inference-time parameters, computation, or latency.

### 4.3. Clean-Setting Evaluation

#### 4.3.1. Controlled Comparison of Metadata-Usage Strategies

We first evaluated the clean-setting utility of structured metadata across the three face-analysis tasks. Table 4 compares four inference-time architectures under the same frozen Face-LLaVA backbone, dataset partitions, training seeds, and optimization protocol. Base provides the visual-only reference, whereas Naive Fusion, Gate Only, and QGFace-LLaVA represent direct, gated, and quality-aware controlled metadata integration, respectively. QGFace-LLaVA + CMRR is not included as a separate inference-time architecture because CMRR modifies only the training objective of QGFace-LLaVA. Its contribution is evaluated separately in the subsequent robustness, internal-control, natural-error, and component-level analyses, where both Clean and imperfect-metadata results are reported.

Under the Clean condition with unperturbed MiVOLO-estimated metadata, QGFace-LLaVA achieves the strongest performance among the four inference-time architectures compared in Table 4 on FER2013 and CelebA-40. Its FER2013 accuracy reaches 52.90 ± 0.78%, exceeding Base, Naive Fusion, and Gate Only, while its CelebA-40 mAcc reaches 91.11 ± 0.05%. The CelebA-40 improvement is modest in absolute magnitude but consistent across training seeds. These results indicate that task-aware quality estimation and controlled residual fusion can provide more consistent clean-setting benefits than direct concatenation or gating without explicit quality estimation.

A different pattern is observed on UTKFace. Gate Only obtains the lowest clean-setting MAE of 3.963 ± 0.131 years, whereas QGFace-LLaVA achieves 5.621 ± 0.098 years while still improving upon Base and Naive Fusion. This contrast motivates the subsequent robustness analyses, which examine whether the stronger clean-setting benefit of Gate Only is maintained under corrupted, missing, and shuffled metadata.

**Figure 4:**
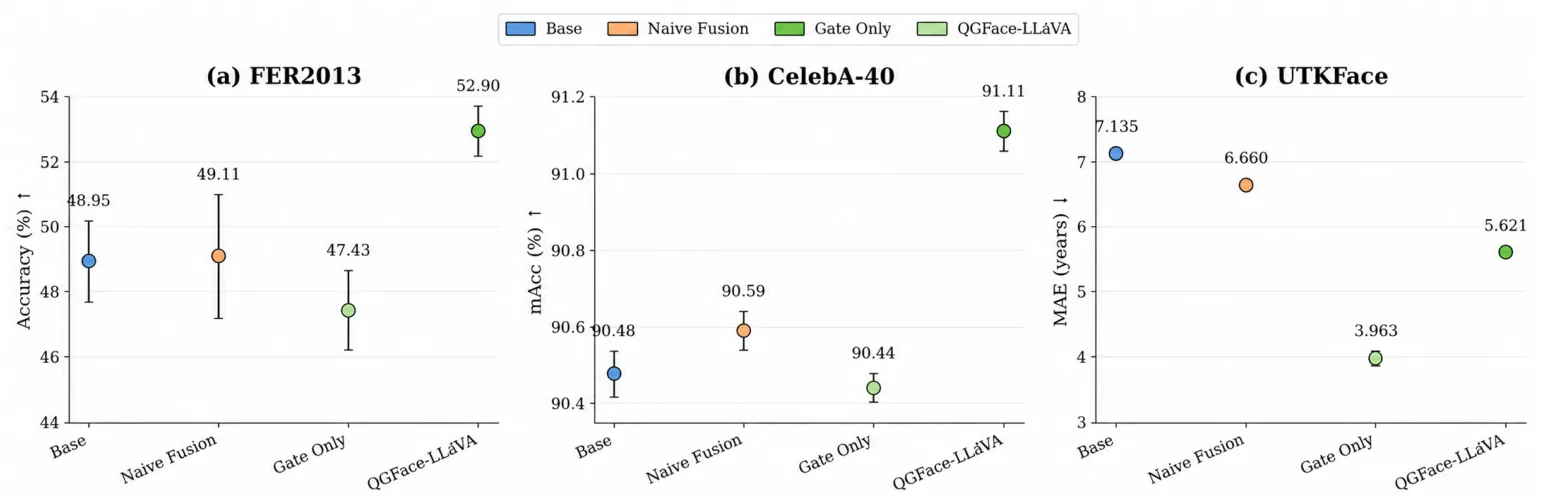
Clean-setting performance of the controlled metadata-usage architectures across FER2013, CelebA-40, and UTKFace. Markers report the mean over three training seeds, and error bars indicate standard deviation. Among the four architectures shown, QGFace-LLaVA achieves the best mean performance on FER2013 and CelebA-40, whereas Gate Only obtains the lowest mean MAE on UTKFace.

Figure 4 provides a visual summary of the task-dependent clean-setting patterns reported in Table 4.

**Figure 5:**
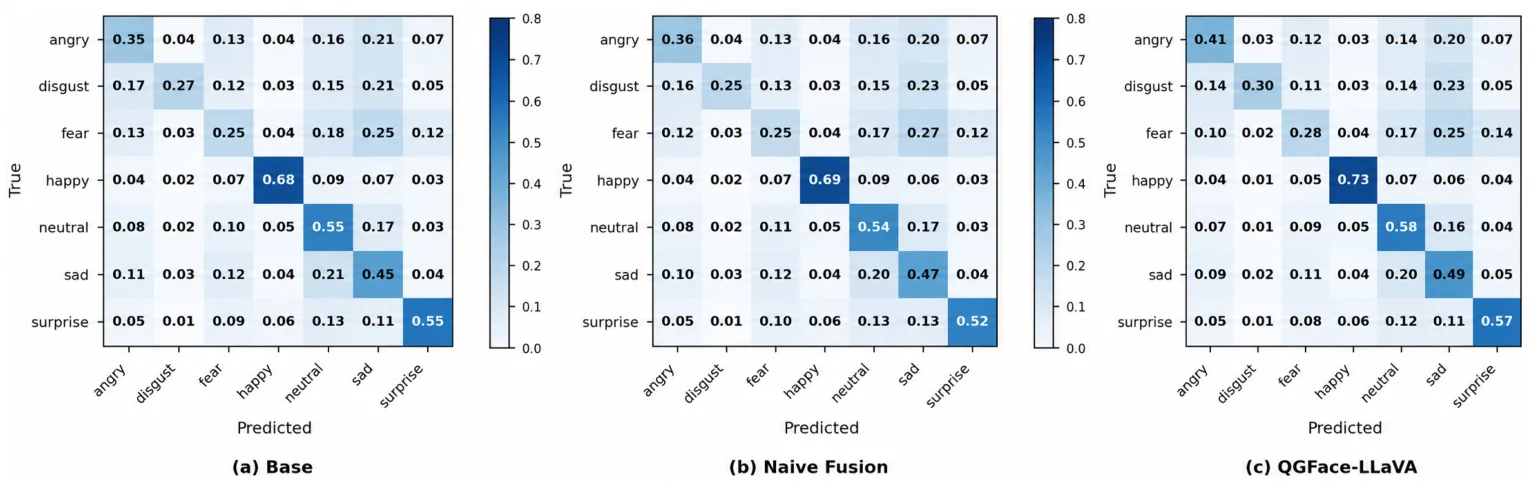
Mean row-normalized confusion matrices on FER2013 under the Clean metadata condition for (**a**) Base, (**b**) Naive Fusion, and (**c**) QGFace-LLaVA. Confusion matrices were computed separately for the checkpoints trained with seeds 42, 2024, and 3407. Each matrix was row-normalized, and the three seed-specific matrices were then averaged element-wise. Each entry represents the mean proportion of samples from a ground-truth class assigned to the corresponding predicted class. Compared with Base and Naive Fusion, QGFace-LLaVA exhibits stronger diagonal concentration for several expression categories, although confusion remains among visually similar expressions.

Figure 5 provides a class-level comparison of three selected architectures on FER2013, representing visual-only prediction, direct metadata fusion, and the proposed quality-aware controlled fusion. QGFace-LLaVA exhibits stronger diagonal concentration for several expression classes than Base and Naive Fusion, although residual confusion remains between visually similar expressions.

#### 4.3.2. Comparison with Recent and Representative Methods

We next compared the core QGFace-LLaVA architecture with representative face-analysis methods reproduced using the fixed dataset partitions and evaluation samples described in Section 4.1. The comparison included facial-expression-recognition methods on FER2013, unified facial-attribute models on CelebA-40, and representative age-estimation or unified face-analysis models on UTKFace. To assess the clean-setting competitiveness of the proposed inference architecture itself, Table 5 reports the core QGFace-LLaVA configuration without CMRR. The contribution of CMRR is evaluated separately in the subsequent robustness, internal-control, natural-error, and component-level analyses, which include both Clean and imperfect-metadata conditions.

External methods were evaluated on the same dataset partitions and final evaluation samples while retaining their official model-specific preprocessing, objectives, and optimization protocols. The comparison therefore evaluates clean-setting competitiveness rather than constituting a controlled architectural ablation.

Table 5 shows that QGFace-LLaVA is competitive with representative external methods but is not uniformly superior to specialized architectures. It approaches ViT for Small-Size Datasets on FER2013 and CORAL-CNN on UTKFace while remaining below the strongest reproduced methods on FER2013 and CelebA-40. These results position QGFace-LLaVA as a unified MLLM-based face-analysis architecture whose primary contribution lies in reliability-aware metadata integration across heterogeneous tasks rather than uniformly state-of-the-art clean-setting performance. The contribution of CMRR is therefore examined separately through the subsequent robustness and mechanism analyses, with particular emphasis on imperfect-metadata conditions where reliability control becomes central.

#### 4.3.3. Backbone Ablation

To assess the contribution of the Face-LLaVA representation, we replaced it with two conventional pretrained visual backbones, ResNet-50 and ViT-B/16, while retaining the same metadata encoder, quality estimator, controlled-fusion module, prediction heads, dataset partitions, training seeds, and optimization protocol. All backbones were frozen, and their output features were projected into the same 256-dimensional fusion space. Ablation used the core QGFace-LLaVA architecture without CMRR so that differences primarily reflected the pretrained backbone representation rather than the additional training-stage regularizer.

All backbones were frozen and evaluated using the same metadata-processing, fusion, and prediction modules. The results are reported as mean ± standard deviation over three seeds.

As shown in Table 6, Face-LLaVA achieves the best mean performance across all three tasks. Relative to ResNet-50, it improves FER2013 accuracy by 4.19 percentage points and CelebA-40 mAcc by 1.13 percentage points while reducing UTKFace MAE by 0.930 years. Relative to ViT-B/16, the corresponding gains are 2.21 percentage points, 0.38 percentage points, and 0.242 years.

These results support Face-LLaVA as the shared representation backbone and suggest that its pretrained visual–language features transfer effectively across classification, multi-label recognition, and regression. However, the backbone comparison does not isolate the source of robustness under imperfect metadata because the quality-aware fusion architecture was held constant. The contributions of quality estimation, gated residual correction, and CMRR are therefore examined separately through the degradation and component-level ablation experiments.

Overall, the clean-setting results show that the core QGFace-LLaVA architecture improves upon simpler metadata-usage strategies on FER2013 and CelebA-40, remains competitive with representative external methods, and benefits from the transferable representation provided by Face-LLaVA. The different pattern observed on UTKFace further motivates evaluation of whether clean-setting gains remain stable when metadata quality or image–metadata correspondence is impaired. The following sections therefore examine the same methods under degraded, missing, naturally erroneous, and semantically mismatched metadata.

### 4.4. Robustness and Mechanism Analysis Under Imperfect Metadata

This section evaluates the core QGFace-LLaVA architecture and its CMRR-enhanced training configuration under corrupted, missing, semantically mismatched, and naturally erroneous metadata. Detailed degradation and progressive-corruption analyses are conducted on CelebA-40 and UTKFace, representing the moderate- and high-alignment settings, while FER2013 is retained in the Clean–Shuffled internal-control and component-level analyses.

#### 4.4.1. Robustness Under Noisy, Missing, and Shuffled Metadata

Table 7 and Table 8 evaluate metadata-assisted methods under the Noisy60, Missing60, and Shuffled conditions on CelebA-40 and UTKFace. These datasets were selected for the detailed degradation analysis because their demographic metadata have moderate or direct relevance to the target tasks. Base is omitted because it receives no metadata and therefore remains unchanged across these conditions. On CelebA-40, the comparison focuses on direct fusion, quality-aware controlled fusion, and CMRR-enhanced training. Gate Only is additionally reported on UTKFace because its strong clean-setting performance provides the clearest case for examining the trade-off between metadata utility and robustness.

Values are means over three training seeds. Direction-aware degradation and MDI are calculated from the reported mean performance.

On CelebA-40, QGFace-LLaVA exhibits lower sensitivity to all three imperfect-metadata conditions than Naive Fusion. Its MDI decreases from 0.250 to 0.050 percentage points, while CMRR-enhanced training further reduces it to 0.020 percentage points. The smaller degradations under Noisy60 and Missing60 similarly indicate that quality-aware regulation limits the influence of degraded or unavailable metadata, with CMRR providing an additional stabilizing effect.

The contrast is more pronounced on UTKFace. Although Gate Only achieves the lowest Clean MAE, it exhibits substantially greater degradation under Noisy60, Missing60, and Shuffled metadata than the other methods. QGFace-LLaVA limits the shuffled-metadata MDI to 0.323 years, and CMRR further reduces it to 0.152 years. Naive Fusion shows less degradation than Gate Only but retains higher absolute MAE than the quality-aware configurations. These results establish clear differences in metadata dependence, which are examined jointly with clean-setting gain in Section 4.5.

#### 4.4.2. Progressive Metadata Corruption

To evaluate sensitivity to increasing corruption severity, the clean-selected checkpoints were tested under the nested Noisy20, Noisy40, Noisy60, and Noisy80 conditions defined in Section 4.2.3. Table 9 and Table 10 report the resulting performance trajectories. Max Δ denotes the largest direction-aware degradation from the clean condition across the four corruption levels.

Values are means over three training seeds. Max Δ is the largest direction-aware degradation from the Clean condition across Noisy20, Noisy40, Noisy60, and Noisy80.

On CelebA-40, performance decreases approximately monotonically as corruption severity increases. The maximum degradation is 0.140 percentage points for Naive Fusion, 0.070 for QGFace-LLaVA, and 0.040 for QGFace-LLaVA + CMRR. This pattern shows that the core reliability-control architecture reduces sensitivity to progressive corruption and that CMRR provides an additional stabilizing effect.

On UTKFace, Gate Only deteriorates from 3.963 years under the Clean condition to 8.123 years under Noisy80, producing the steepest degradation trajectory. QGFace-LLaVA maintains a lower absolute MAE than Naive Fusion at every corruption level, although its Max Δ of 0.212 years is slightly larger than the 0.158 years of Naive Fusion. CMRR reduces the Max Δ of QGFace-LLaVA to 0.085 years, indicating greater stability as the proportion of corrupted metadata increases.

#### 4.4.3. Internal Metadata Control and Quality-Score Validation

We first examined whether the internal quality score and fusion gate responded to semantic mismatch and then evaluated whether the learned quality score distinguished reliable from unreliable metadata at the sample level. Table 11 and Table 12 report the condition-level and sample-level analyses, respectively.

For each training seed, quality scores and gate magnitudes were first averaged over all samples in the fixed test partition under each metadata condition. The reported values were then obtained by averaging the three seed-level means from checkpoints trained with seeds 42, 2024, and 3407.

Table 11 shows that both the quality score and mean gate magnitude decrease under Shuffled metadata across all three datasets, with consistently larger reductions after CMRR-enhanced training. On UTKFace, for example, the quality score decreases from 0.78 to 0.35 and the mean gate magnitude decreases from 0.41 to 0.14 after CMRR-enhanced training, compared with decreases from 0.77 to 0.48 and from 0.43 to 0.22, respectively, for the core architecture. These patterns indicate that CMRR strengthens the suppression of semantically mismatched metadata while retaining substantial metadata use under the Clean condition.

To evaluate sample-level validity, we used the QGFace-LLaVA + CMRR checkpoints to compare quality scores between reliable and unreliable metadata and to measure their discriminative and associative relationships with metadata correctness.

Reliable metadata were defined as MiVOLO gender estimates that agreed with the ground-truth male attribute on CelebA-40 and as estimates with an absolute age error of no greater than 10 years on UTKFace. Gender disagreement and age errors greater than 10 years were treated as unreliable metadata. For the Noisy60 analyses, perturbed and unperturbed samples were treated as unreliable and reliable, respectively. AUROC was calculated using $q_{t}$ as the discrimination score, with reliable metadata treated as the positive class. Here, $r_{rb}$ denotes rank-biserial correlation; Spearman’s ρ was calculated against absolute age-estimation error for UTKFace natural errors and against binary corruption status for Noisy60. The four quality-score validation *p*-values were adjusted using the Holm procedure.

Table 12 shows consistently higher mean quality scores for reliable metadata than for unreliable metadata. Under naturally occurring errors, AUROC reaches 0.72 for CelebA-40 gender correctness and 0.81 for UTKFace age reliability. The positive rank-biserial association on CelebA-40 indicates that correct gender estimates generally receive higher quality scores, whereas the negative Spearman correlation on UTKFace indicates that larger absolute age-estimation errors are associated with lower predicted reliability.

Under Noisy60, AUROC reaches 0.81 on CelebA-40 and 0.88 on UTKFace. The negative correlations with binary corruption status indicate that perturbed samples generally receive lower quality scores than unperturbed samples. All four quality-score validation comparisons remain statistically significant after Holm correction. The lower AUROC values under naturally occurring errors suggest that visually plausible estimator errors are more difficult to distinguish than controlled synthetic corruption.

On the fixed UTKFace test partition, 4180 samples satisfied the reliable-metadata criterion and 642 samples were classified as unreliable. Using the QGFace-LLaVA + CMRR configuration, the mean gate magnitude decreased from 0.4233 for reliable metadata to 0.2337 for unreliable metadata. This exploratory gate-magnitude comparison was analyzed separately and was not included in the four-comparison Holm correction applied to the quality-score validation results. The observed reduction indicates that naturally erroneous age metadata were generally assigned weaker fusion influence.

#### 4.4.4. Component-Level Ablation

We evaluated the architectural and training-stage reliability controls under the Clean and Shuffled metadata conditions. Full denotes QGFace-LLaVA trained with CMRR, whereas Full w/o CMRR corresponds to the core QGFace-LLaVA architecture. The Full w/o quality-aware regulation variant disables both task-aware quality estimation and quality-guided metadata calibration. The Full w/o fusion gate variant removes the adaptive gate and directly injects the calibrated metadata residual. All remaining modules and applicable loss terms, together with the data partitions, training seeds, optimization settings, and evaluation conditions, were held fixed.

These component-level variants were obtained by removing individual components from the full configuration and should not be confused with the independently constructed Gate Only baseline described in Section 4.2.1. When a component was removed, any CMRR term that directly depended on that component was disabled accordingly, while all remaining applicable CMRR terms were retained.The component-level ablation results under the Clean and Shuffled metadata conditions are summarized in Table 13.

**Table 13:** Component-level ablation under the Clean and Shuffled metadata conditions.

Dataset	Variant	Metric	Clean	Shuffled	MDI ↓
FER2013	Full	Accuracy (%) ↑	53.11 ± 0.88	52.97 ± 1.10	0.14
	Full w/o CMRR	Accuracy (%) ↑	52.90 ± 0.78	52.46 ± 1.23	0.44
	Full w/o quality-aware regulation	Accuracy (%) ↑	51.63 ± 1.13	51.02 ± 1.37	0.61
	Full w/o fusion gate	Accuracy (%) ↑	51.35 ± 1.21	50.17 ± 0.77	1.18
CelebA-40	Full	mAcc (%) ↑	91.15 ± 0.03	91.13 ± 0.05	0.02
	Full w/o CMRR	mAcc (%) ↑	91.11 ± 0.05	91.06 ± 0.13	0.05
	Full w/o quality-aware regulation	mAcc (%) ↑	90.86 ± 0.12	90.55 ± 0.09	0.31
	Full w/o fusion gate	mAcc (%) ↑	90.91 ± 0.11	90.34 ± 0.09	0.57
UTKFace	Full	MAE (years) ↓	5.587 ± 0.188	5.739 ± 0.247	0.152
	Full w/o CMRR	MAE (years) ↓	5.621 ± 0.098	5.944 ± 0.178	0.323
	Full w/o quality-aware regulation	MAE (years) ↓	5.804 ± 0.163	6.612 ± 0.272	0.808
	Full w/o fusion gate	MAE (years) ↓	5.416 ± 0.211	7.117 ± 0.199	1.701

Note: ↑ indicates that higher values are better, whereas ↓ indicates that lower values are better.

Clean and Shuffled results are reported as mean ± standard deviation over three seeds. MDI is calculated from the reported mean values and is expressed in percentage points for FER2013 and CelebA-40 and in years for UTKFace.

Removing CMRR produces only small changes in Clean performance but consistently increases sensitivity to Shuffled metadata. MDI increases from 0.14 to 0.44 percentage points on FER2013, from 0.02 to 0.05 percentage points on CelebA-40, and from 0.152 to 0.323 years on UTKFace. These results indicate that CMRR primarily improves robustness to semantic metadata mismatch rather than substantially changing clean-setting performance.

Removing quality-aware regulation further increases MDI to 0.61 percentage points on FER2013, 0.31 percentage points on CelebA-40, and 0.808 years on UTKFace. Removing the fusion gate produces the largest mismatch sensitivity, with corresponding MDI values of 1.18 percentage points, 0.57 percentage points, and 1.701 years. On UTKFace, removing the fusion gate reduces Clean MAE from 5.587 to 5.416 years but increases Shuffled MAE from 5.739 to 7.117 years, confirming that the adaptive gate is central to limiting harmful metadata influence under semantic mismatch. Overall, quality-aware regulation and gated residual correction constitute the principal architectural reliability controls, while CMRR provides an additional training-stage robustness benefit.

#### 4.4.5. Naturally Occurring Metadata Errors and Failure Cases

The Clean condition retains the original, unperturbed MiVOLO estimates and may therefore still contain naturally occurring estimation errors. Although the synthetic Noisy conditions provide controlled corruption severity, they may not fully represent the errors produced by a real metadata estimator. We therefore conducted a post hoc diagnostic evaluation of naturally occurring MiVOLO errors on CelebA-40 and UTKFace. For CelebA-40, the diagnostic subset comprised test samples for which the MiVOLO-estimated gender disagreed with the ground-truth gender label represented by the Male attribute. For UTKFace, the diagnostic subset comprised test samples with an absolute MiVOLO age-estimation error greater than 10 years, following the natural-reliability criterion defined in Section 4.4.3.

For this post hoc diagnostic analysis, ground-truth annotations were used only to identify test samples containing naturally occurring MiVOLO errors and were never provided to the model as inputs. Each natural-error condition was paired with an erroneous-channel-suppressed condition evaluated using the same trained checkpoint and identical test samples. Here, erroneous-channel suppression was operationalized by setting the identified erroneous metadata channel and its associated confidence score to zero rather than removing the channel from the input vector. On the CelebA-40 wrong-gender subset, the estimated-gender channel and gender-confidence score were set to zero, while the estimated-age channel and age-confidence score were retained. On the UTKFace subset with an absolute MiVOLO age-estimation error greater than 10 years, the estimated-age channel and age-confidence score were set to zero, while the estimated-gender channel and gender-confidence score were retained. Because valid metadata from the other demographic channel remained available, the overall availability indicator was left unchanged. No additional training, checkpoint selection, or condition-specific optimization was performed.The post hoc diagnostic results for naturally occurring metadata errors and erroneous-channel suppression are summarized in Table 14.

**Table 14:** Post hoc diagnostic evaluation of naturally occurring MiVOLO metadata errors and erroneous-channel suppression.

Dataset/Error Subset	Method	Original	Suppressed	Error-Induced Harm ↓
CelebA-40/Wrong-Gender Subset mAcc (%) ↑	Naive Fusion	89.45 ± 0.12	89.71 ± 0.17	0.26 ± 0.16
	QGFace-LLaVA	89.97 ± 0.08	90.11 ± 0.11	0.14 ± 0.13
	QGFace-LLaVA + CMRR	90.33 ± 0.06	90.45 ± 0.09	0.12 ± 0.07
UTKFace/Absolute Age Error > 10 Years MAE (years) ↓	Naive Fusion	9.62 ± 0.26	9.19 ± 0.31	0.43 ± 0.21
	QGFace-LLaVA	8.79 ± 0.18	8.47 ± 0.23	0.32 ± 0.17
	QGFace-LLaVA + CMRR	8.18 ± 0.13	8.06 ± 0.15	0.12 ± 0.11

Note: ↑ indicates that higher values are better, whereas ↓ indicates that lower values are better.

Original denotes performance obtained using the unmodified MiVOLO metadata on the selected natural-error subset. Suppressed denotes performance after setting the identified erroneous metadata channel and its associated confidence score to zero while retaining the other demographic channel and the overall availability indicator. The results are reported as mean ± standard deviation over seeds 42, 2024, and 3407. Error-induced harm was calculated separately for each seed as Suppressed − Original for CelebA-40 mAcc and as Original − Suppressed for UTKFace MAE. Positive values therefore indicate performance improvement after suppressing the erroneous metadata channel. Error-induced harm is reported in percentage points for CelebA-40 and in years for UTKFace.

On CelebA-40, zeroing the erroneous gender channel and its associated confidence score increases mAcc by 0.26 percentage points for Naive Fusion, 0.14 percentage points for QGFace-LLaVA, and 0.12 percentage points for QGFace-LLaVA + CMRR. On UTKFace, zeroing the erroneous age channel and its associated confidence score reduces MAE by 0.43, 0.32, and 0.12 years, respectively. The positive mean error-induced harm values indicate that retaining naturally erroneous metadata was generally more detrimental than zeroing the identified erroneous channel and its confidence score on these diagnostic subsets. Moreover, the progressively smaller harm observed for Naive Fusion, QGFace-LLaVA, and QGFace-LLaVA + CMRR is consistent with reduced sensitivity to naturally erroneous auxiliary information after quality-aware regulation and CMRR-enhanced training.

Although QGFace-LLaVA + CMRR reduced mean error-induced harm on the diagnostic subset, some failures recurred across multiple training seeds. We screened all samples in the fixed UTKFace test partition with an absolute MiVOLO age-estimation error greater than 10 years. For each sample, the absolute prediction errors of Base and Full were calculated separately using checkpoints trained with seeds 42, 2024, and 3407. A sample qualified when Full had a larger mean absolute error than Base and performed worse than Base in at least two of the three seeds. Qualifying samples were ranked by the difference between the mean absolute errors of Full and Base, and the four samples with the largest differences are reported in Table 15. Case selection was performed independently of the quality score, gate magnitude, and subsequent failure-mode interpretation.

GT denotes ground-truth age, and Full denotes QGFace-LLaVA + CMRR. In the “Age (GT/MiVOLO)” column, the two values represent the ground-truth age and MiVOLO-estimated age, respectively. Abs. Age Error is the absolute difference between these two ages. Ages, absolute age errors, and Base and Full predictions are reported in years. Base and Full predictions, quality scores, and gate magnitudes are reported as mean ± standard deviation across checkpoints trained with seeds 42, 2024, and 3407.

The four cases reveal distinct metadata-control failure mechanisms. In Case 1, the mean quality score remains relatively high despite a 22-year metadata error, indicating reliability overestimation. In Case 2, the Full prediction shifts toward the underestimated MiVOLO age and farther from the ground truth, demonstrating a metadata-induced directional shift. Case 3 exhibits the highest mean gate magnitude among the four cases despite a 20-year metadata error, indicating insufficient suppression of unreliable age information. In Case 4, the Full prediction moves away from both the ground truth and the MiVOLO estimate, suggesting an adverse interaction between the metadata residual and visual representation rather than a simple directional pull toward the erroneous metadata. These cases complement the aggregate results by showing that residual failures may arise from quality overestimation, insufficient gating, or harmful feature-level interactions even when metadata-related harm is reduced on average.

### 4.5. Metadata Utility Boundary and the Performance–Robustness Trade-Off

We next examined whether clean-setting improvements are consistently associated with lower sensitivity to semantic metadata mismatch by jointly analyzing clean gain and MDI on CelebA-40 and UTKFace. Clean gain was calculated relative to Base: for CelebA-40, it was defined as the metadata-assisted mAcc minus the Base mAcc; for UTKFace, it was defined as the Base MAE minus the metadata-assisted MAE. Positive values therefore consistently indicate improved clean-setting performance. MDI follows the definition in Section 4.2.3. Because the two datasets use different metrics, clean gain and MDI are interpreted only within the same dataset.

Table 16 shows that clean-setting gain and semantic-mismatch sensitivity are not monotonically related. On CelebA-40, quality-aware regulation improves clean performance while reducing MDI relative to Naive Fusion. On UTKFace, Gate Only occupies a high-gain, high-dependence regime, whereas QGFace-LLaVA and its CMRR-enhanced configuration achieve a more balanced utility–robustness profile. These results indicate that the magnitude of the clean-setting benefit alone is insufficient to characterize reliable metadata use.

**Figure 6:**
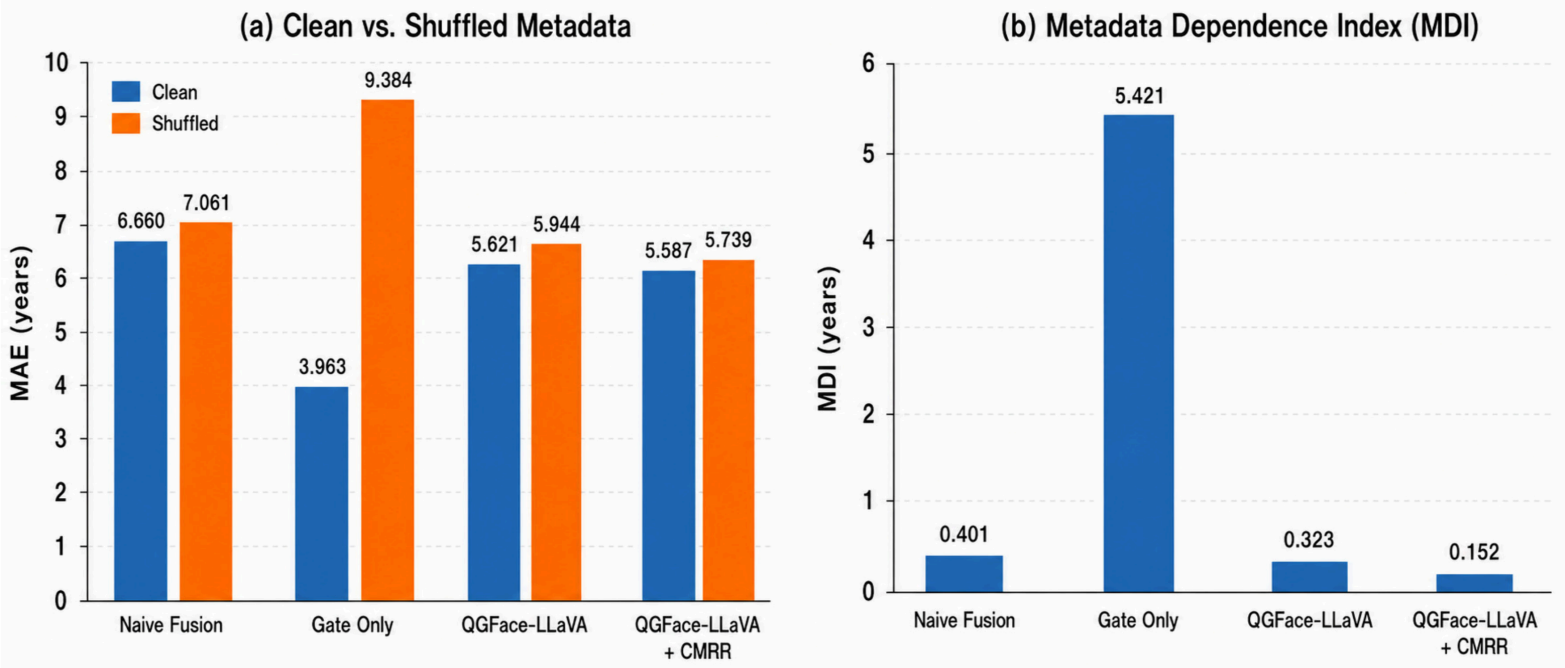
Performance–robustness trade-off on UTKFace under the Clean and Shuffled metadata conditions. (**a**) Mean MAE under the two metadata conditions. (**b**) Metadata Dependence Index (MDI), computed as the absolute difference between the mean MAE under the Clean and Shuffled conditions. Gate Only achieves the lowest Clean MAE but the highest MDI, whereas QGFace-LLaVA + CMRR achieves the lowest MDI. Clean and Shuffled MAE values are reported as means over checkpoints trained with seeds 42, 2024, and 3407, and MDI is computed from these mean values.

Figure 6 further visualizes the utility–robustness contrast on UTKFace.

Together, Table 16 and Figure 6 define a practical metadata-utility boundary: auxiliary information should be evaluated jointly in terms of clean-setting benefit and semantic-mismatch sensitivity.

### 4.6. Prompt-Level Counterfactual Sensitivity

To complement the vector-level metadata perturbation experiments, we evaluated the sensitivity of CelebA-40 predictions to textual demographic cues presented in the prompt. The same trained checkpoints and test images were evaluated under four prompt conditions: No-Metadata Prompt, Clean-Metadata Prompt, Counterfactual-Metadata Prompt, and Low-Reliability-Metadata Prompt. The image input, attribute query, model parameters, task-specific prediction head, and structured five-channel metadata vector supplied to the metadata-fusion module were held fixed across all four conditions; only the metadata-related textual content of the prompt was modified. No additional training, checkpoint selection, or prompt-specific optimization was performed.

The No-Metadata Prompt condition omitted all demographic descriptions from the prompt. The Clean-Metadata Prompt condition included the original age- and gender-related information estimated by MiVOLO. The Counterfactual-Metadata Prompt condition replaced these demographic descriptions with semantically inconsistent values. The Low-Reliability-Metadata Prompt condition retained the same demographic content as the Clean-Metadata Prompt condition but explicitly described the information as having low reliability. Apart from these metadata-related textual statements, all other prompt content and all structured model inputs remained identical across the four conditions.

For image i, attribute a, and prompt condition c, let $z_{i,a}^{\left(c\right)}$ denote the scalar logit produced by the CelebA-40 task-specific prediction head. The No-Metadata Prompt condition is denoted by c=0. Prompt sensitivity relative to the No-Metadata Prompt condition was defined as $S_{i,a}^{\left(c\right)}=\left|z_{i,a}^{\left(c\right)}-z_{i,a}^{\left(0\right)}\right|$ A larger value of $S_{i,a}^{\left(c\right)}$ indicates a greater absolute change in the corresponding attribute logit relative to the No-Metadata Prompt condition.

**Figure 7:**
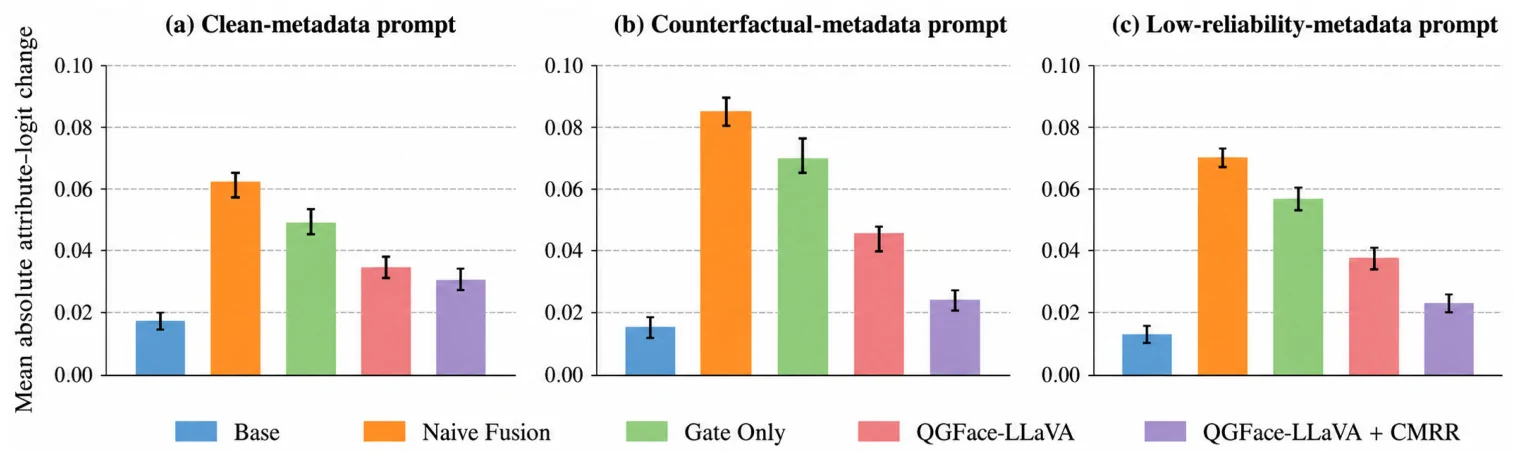
Prompt-conditioned prediction sensitivity on CelebA-40. Sensitivity is the absolute change in attribute-specific task-head logits relative to the No-Metadata Prompt, averaged across image–attribute pairs and three training seeds. Error bars indicate standard deviation. Lower sensitivity to counterfactual and low-reliability metadata prompts indicates greater resistance to misleading demographic cues.

For each training seed, the absolute prompt-sensitivity values were first averaged over all evaluated image–attribute pairs. Figure 7 reports the mean of the three seed-level averages obtained using seeds 42, 2024, and 3407, with error bars indicating the standard deviation across the three seed-level means. Under the Counterfactual-Metadata Prompt and Low-Reliability-Metadata Prompt conditions, lower sensitivity indicates stronger resistance to unreliable or potentially misleading textual demographic cues. Under the Clean-Metadata Prompt condition, sensitivity reflects the model’s responsiveness to potentially useful textual metadata and is therefore not interpreted independently as either beneficial or detrimental.

Figure 7 shows clear differences in prompt-level metadata sensitivity. Under the Counterfactual-Metadata Prompt and Low-Reliability-Metadata Prompt conditions, Naive Fusion produces the largest absolute changes in the attribute-specific task-head logits, while Gate Only provides partial suppression. QGFace-LLaVA exhibits lower sensitivity under both imperfect-prompt conditions, indicating greater stability against unreliable or potentially misleading textual demographic cues.

QGFace-LLaVA + CMRR produces the smallest absolute logit changes under the two imperfect-prompt interventions. This pattern is consistent with its lower MDI in Table 7 and suggests that its robustness advantage is also reflected in greater stability under prompt-level textual metadata interventions. Because the structured five-channel metadata vector was held fixed throughout this analysis, these results should be interpreted as a complementary prompt-sensitivity probe rather than as a direct evaluation of the structured-metadata quality estimator or fusion gate.

### 4.7. Statistical Analysis of Selected Comparisons

To assess the statistical reliability of the principal performance and robustness differences, we conducted six targeted paired comparisons covering the main clean-setting, shuffled-metadata, and naturally occurring metadata-error conditions. Identical test samples were used for all methods within each comparison. A cross-seed paired permutation test was performed jointly across checkpoints trained with seeds 42, 2024, and 3407. Sample-level performance differences were first calculated within each seed, and the observed statistic was defined as their mean across the three seeds and matched test samples. During permutation, method labels were exchanged at the sample level, with the same exchange applied simultaneously to all three seed-specific outputs, thereby preserving the dependence induced by evaluating multiple checkpoints on the same samples. For CelebA-40, all 40 attribute predictions from one image were treated as a single paired unit, whereas UTKFace comparisons used paired absolute age-prediction errors. Two-sided *p*-values were estimated using 10,000 permutations and adjusted across the six comparisons using the Holm procedure. Performance gains were calculated as the mean of the three seed-specific paired differences, and 95% confidence intervals were estimated using 10,000 paired bootstrap replicates while retaining all seed-specific outputs for each resampled test sample. Positive gain values indicate better performance by the first method.The results of the six selected paired comparisons are summarized in Table 17.

**Table 17:** Statistical analysis of selected paired performance and robustness comparisons.

Dataset/Condition	Comparison	Mean Gain ↑	95% CI	Holm-Adjusted *p*-Value
FER2013/Clean	QGFace-LLaVA vs. Base	+3.95 pp	[+2.51, +5.37] pp	0.018
CelebA-40/Clean	QGFace-LLaVA vs. Naive Fusion	+0.52 pp	[+0.39, +0.67] pp	0.023
CelebA-40/Shuffled	QGFace-LLaVA + CMRR vs. Naive Fusion	+0.79 pp	[+0.61, +1.07] pp	0.017
UTKFace/Clean	QGFace-LLaVA vs. Naive Fusion	+1.039 years	[+0.91, +1.27] years	0.032
UTKFace/Shuffled	QGFace-LLaVA + CMRR vs. Gate Only	+3.645 years	[+3.21, +4.07] years	0.013
UTKFace/MiVOLO age error > 10 years	QGFace-LLaVA + CMRR vs. Naive Fusion	+1.44 years	[+1.11, +1.83] years	0.028

Note: ↑ indicates that higher values are better; pp denotes percentage points.

All six selected comparisons remained statistically significant after Holm correction, with adjusted *p*-values ranging from 0.013 to 0.032. Under the Clean condition, QGFace-LLaVA outperformed Base on FER2013 and Naive Fusion on both CelebA-40 and UTKFace. Under Shuffled metadata, QGFace-LLaVA + CMRR outperformed Naive Fusion on CelebA-40 and Gate Only on UTKFace. The CMRR-enhanced configuration also reduced MAE relative to Naive Fusion on the UTKFace subset with MiVOLO age-estimation errors greater than 10 years.

Statistical significance should nevertheless be interpreted together with effect magnitude. The 0.52-percentage-point gain on CelebA-40 under Clean metadata is statistically reliable but modest in practical magnitude, whereas the 3.645-year MAE reduction on UTKFace under Shuffled metadata is substantial in both statistical and practical terms. These results show that statistical reliability does not necessarily imply a large practical benefit and should therefore be considered together with the reported performance gains and confidence intervals.

### 4.8. Qualitative Attribution Visualization

**Figure 8:**
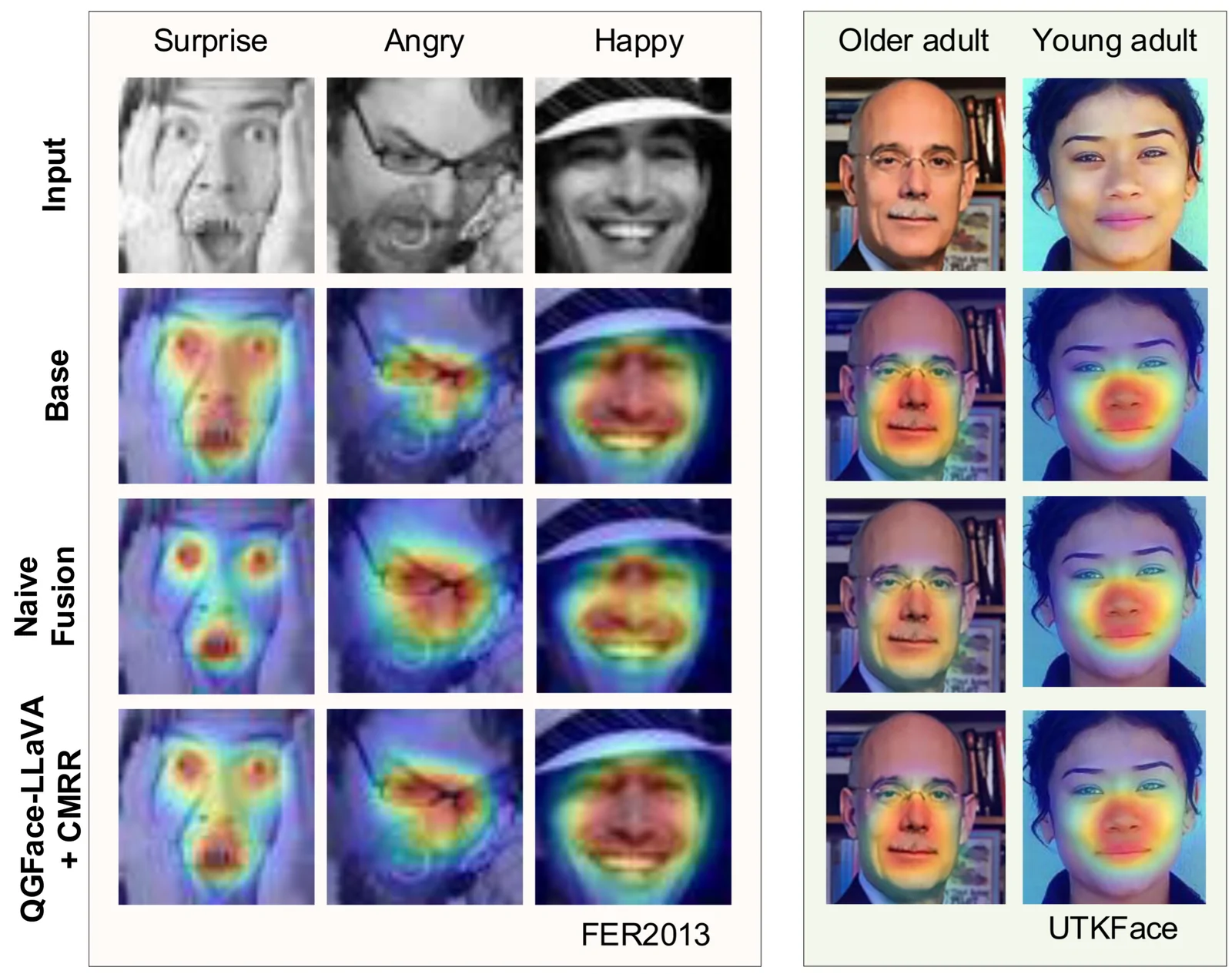
Qualitative Grad-CAM attribution visualizations on FER2013 and UTKFace. The first row shows the original input images, followed by the attribution maps produced by Base, Naive Fusion, and QGFace-LLaVA + CMRR. The FER2013 samples represent surprise, anger, and happiness, whereas the UTKFace samples represent an older adult and a young adult. Across the selected examples, QGFace-LLaVA + CMRR produces more spatially concentrated attribution over task-relevant facial regions than the compared strategies.Warmer colors indicate higher attribution intensity, whereas cooler colors indicate lower attribution intensity.

To complement the quantitative robustness evaluation, Figure 8 presents Grad-CAM-based attribution maps [67] for selected FER2013 and UTKFace test samples. The comparison includes Base, Naive Fusion, and QGFace-LLaVA + CMRR. All attribution maps were generated using the corresponding checkpoints trained with seed 2024. For each dataset, the same test samples, target layer, task-specific output targets, preprocessing procedure, and visualization settings were used across the three methods. For FER2013, attribution was computed with respect to the ground-truth class logit, whereas for UTKFace, attribution was computed with respect to the scalar age-regression output. The displayed samples were selected independently of the appearance of the attribution maps, and the same samples and output targets were retained across all compared methods.

Across the illustrated samples, QGFace-LLaVA + CMRR generally maintains attribution around task-relevant facial regions. For FER2013, its responses are concentrated primarily around expression-related regions, particularly the eyes and mouth. For UTKFace, the responses emphasize central facial structures and facial texture patterns relevant to age estimation. Compared with Base and Naive Fusion, the full configuration exhibits more spatially concentrated attribution in the selected examples. These qualitative observations complement the quantitative degradation, metadata-dependence, and natural-error analyses but are not interpreted as standalone evidence of superior robustness.

### 4.9. Discussion of Experimental Findings

Across the three tasks, the results define a task-dependent metadata-utility boundary: structured metadata can improve prediction when they are relevant and reliable, but clean-setting gains alone do not imply robustness under imperfect metadata. Metadata-assisted face-analysis systems should therefore be evaluated jointly in terms of clean utility, absolute performance under degraded or mismatched conditions, and sensitivity to metadata corruption.

The results also distinguish architectural control from training-stage robustness. The core QGFace-LLaVA architecture reduces metadata dependence, whereas CMRR provides additional stability under progressive corruption and semantic mismatch. Internal-score validation, component-level ablation, naturally occurring metadata-error analysis, and statistical testing support this interpretation. Nevertheless, the identified failure cases show that quality overestimation, insufficient gate suppression, and adverse residual interactions remain unresolved. From a biomimetic perspective, these findings support selective and reliability-dependent cue use rather than fixed or unconditional fusion.

## 5. Limitations and Future Work

This study has several limitations and directions for future work. First, the present work focuses on compact structured metadata, including demographic cues, confidence scores, and availability indicators. Future studies may incorporate richer auxiliary signals, such as facial landmarks, image quality indicators, acquisition conditions, or contextual descriptions, to further examine metadata utility under more diverse conditions.

Second, although the experiments cover three representative face analysis tasks, broader evaluation across additional datasets, demographic distributions, annotation protocols, and real-world metadata acquisition pipelines would further validate the generality of the proposed framework.

Third, the present analysis mainly focuses on predictive performance, robustness, and metadata dependence. Future work may further investigate fairness, calibration, uncertainty estimation, and subgroup-level reliability, especially because structured metadata such as age and gender may introduce demographic shortcuts or biased decision patterns if not properly controlled. In QGFace-LLaVA, such metadata are therefore used as reliability-controlled auxiliary cues rather than dominant prediction evidence.

Finally, although CMRR improves robustness by exposing the model to counterfactual metadata during training, the present implementation mainly considers shuffled or semantically mismatched metadata. Future work may explore more diverse counterfactual metadata construction strategies, adaptive reliability thresholds, and real-world metadata conflicts while preserving the reliability advantages of quality-aware controlled fusion.

## 6. Conclusions

This study examined the conditional utility of structured side information in MLLM-based face analysis under imperfect metadata. Across facial expression recognition, facial attribute recognition, and age estimation, the results show that metadata utility depends jointly on task relevance, metadata reliability, and fusion strategy. Clean-setting improvements therefore should not be interpreted as evidence of robustness when metadata are noisy, missing, or mismatched.

QGFace-LLaVA addresses this problem by treating structured metadata as a reliability-controlled auxiliary cue while preserving visual evidence as the primary prediction source. The controlled-fusion architecture reduces metadata dependence, and CMRR provides additional stability under metadata corruption and mismatch. Overall, the findings support selective, reliability-aware cue integration and highlight the need to evaluate metadata-assisted face analysis under both clean and imperfect conditions.

## Figures and Tables

**Table 1:** Conceptual comparison of representative side-information fusion strategies and the proposed framework.

Method	Reliability	Task-Aware Quality	Controlled Fusion	Counterfactual Training	Imperfect-Input Evaluation
Direct concatenation	—	—	—	—	—
FiLM [45]	—	—	✓	—	—
DAFT [46]	—	—	✓	—	—
Credibility-Weighted Mean (CWM) [52]	✓	—	✓	—	✓
QGFace-LLaVA	✓	✓	✓	—	✓
QGFace-LLaVA + CMRR	✓	✓	✓	✓	✓

Note: “✓” indicates that the corresponding characteristic is explicitly incorporated or evaluated, whereas “—” indicates that it is not explicitly incorporated or reported.

**Table 2:** Dataset characteristics, fixed partitions, and evaluation metrics.

Dataset	Task (Alignment)	Train	Validation	Test	Metric
FER2013	Expression classification (low)	28,709	3589	3589	Accuracy (%) ↑
CelebA-40	Multi-label attribute classification (moderate)	162,770	19,867	19,962	mAcc (%) ↑
UTKFace	Age regression (high)	17,355	1929	4822	MAE (years) ↓

Note: ↑ indicates that higher values are better, whereas ↓ indicates that lower values are better.

**Table 3:** Incremental computational overhead of the trainable task-head and metadata-fusion modules.

Method	Trainable Module Parameters (M) ↓	Incremental MFLOPs ↓	Incremental Latency (ms) ↓
Base	1.060	2.118	0.050 ± 0.003
Naive Fusion	1.225	2.447	0.136 ± 0.006
Gate Only	1.226	2.448	0.145 ± 0.006
QGFace-LLaVA (+/− CMRR)	1.231	2.457	0.205 ± 0.010

Note: ↓ indicates that lower values are better.

**Table 4:** Clean-setting performance of controlled metadata-usage strategies over three training seeds.

Dataset	Metric	Base	Naive Fusion	Gate Only	QGFace-LLaVA
FER2013	Accuracy (%) ↑	48.95 ± 1.28	49.11 ± 1.91	47.43 ± 1.24	52.90 ± 0.78
CelebA-40	mAcc (%) ↑	90.48 ± 0.06	90.59 ± 0.05	90.44 ± 0.04	91.11 ± 0.05
UTKFace	MAE (years) ↓	7.135 ± 0.111	6.660 ± 0.092	3.963 ± 0.131	5.621 ± 0.098

Note: ↑ indicates that higher values are better, whereas ↓ indicates that lower values are better.

**Table 5:** Clean-setting comparison with recent and representative reproducible face-analysis methods under the fixed evaluation protocol.

Dataset	Method	Result
FER2013 (Acc. %, ↑)	ViT for Small-Size Datasets [62]	53.21 ± 0.73
	GLRLDLW [63]	59.77 ± 0.97
	QGFace-LLaVA	52.90 ± 0.78
CelebA-40 (mAcc. %, ↑)	Faceptor-Full [17]	91.52 ± 0.07
	FaceXFormer [41]	91.97 ± 0.09
	QGFace-LLaVA	91.11 ± 0.05
UTKFace (MAE, ↓)	AnyFace++ [64]	5.283 ± 0.113
	CORAL-CNN [65]	5.557 ± 0.088
	QGFace-LLaVA	5.621 ± 0.098

Note: ↑ indicates that higher values are better, whereas ↓ indicates that lower values are better.

**Table 6:** Backbone ablation under the same quality-aware metadata-fusion protocol.

Backbone	FER2013 Acc. (%) ↑	CelebA-40 mAcc. (%) ↑	UTKFace MAE (Years) ↓
ResNet-50	48.71 ± 1.38	89.98 ± 0.18	6.551 ± 0.198
ViT-B/16	50.69 ± 0.87	90.73 ± 0.07	5.863 ± 0.103
Face-LLaVA	52.90 ± 0.78	91.11 ± 0.05	5.621 ± 0.098

Note: ↑ indicates that higher values are better, whereas ↓ indicates that lower values are better.

**Table 7:** Robustness and metadata dependence on CelebA-40 measured by mAcc (%).

Method	Clean	Noisy60	Missing60	Shuffled	ΔNoisy60	ΔMissing60	MDI
Naive Fusion	90.59	90.50	90.43	90.34	0.090	0.160	0.250
QGFace-LLaVA	91.11	91.07	91.09	91.06	0.040	0.020	0.050
QGFace-LLaVA + CMRR	91.15	91.13	91.14	91.13	0.020	0.010	0.020

**Table 8:** Robustness and metadata dependence on UTKFace measured by MAE (years).

Method	Clean	Noisy60	Missing60	Shuffled	ΔNoisy60	ΔMissing60	MDI
Naive Fusion	6.660	6.742	6.716	7.061	0.082	0.056	0.401
Gate Only	3.963	6.944	7.402	9.384	2.981	3.439	5.421
QGFace-LLaVA	5.621	5.741	5.802	5.944	0.120	0.181	0.323
QGFace-LLaVA + CMRR	5.587	5.626	5.667	5.739	0.039	0.080	0.152

**Table 9:** Performance under progressive metadata corruption on CelebA-40 measured by mAcc (%).

Method	Clean	Noisy20	Noisy40	Noisy60	Noisy80	Max Δ ↓
Naive Fusion	90.59	90.56	90.53	90.50	90.45	0.14
QGFace-LLaVA	91.11	91.10	91.08	91.07	91.04	0.07
QGFace-LLaVA + CMRR	91.15	91.15	91.14	91.13	91.11	0.04

Note: ↓ indicates that lower values are better.

**Table 10:** Performance under progressive metadata corruption on UTKFace measured by MAE (years).

Method	Clean	Noisy20	Noisy40	Noisy60	Noisy80	Max Δ ↓
Naive Fusion	6.660	6.690	6.718	6.742	6.818	0.158
Gate Only	3.963	4.926	5.887	6.944	8.123	4.160
QGFace-LLaVA	5.621	5.666	5.708	5.741	5.833	0.212
QGFace-LLaVA + CMRR	5.587	5.599	5.613	5.626	5.672	0.085

Note: ↓ indicates that lower values are better.

**Table 11:** Internal quality and gating responses under the Clean and Shuffled metadata conditions.

Dataset	Method	Mean Quality Score $q_{t}$ (Clean → Shuffled)	Mean Gate Magnitude (Clean → Shuffled)
FER2013	QGFace-LLaVA	0.42 → 0.27	0.16 → 0.09
FER2013	QGFace-LLaVA + CMRR	0.43 → 0.20	0.15 → 0.06
CelebA-40	QGFace-LLaVA	0.61 → 0.41	0.26 → 0.15
CelebA-40	QGFace-LLaVA + CMRR	0.63 → 0.34	0.25 → 0.10
UTKFace	QGFace-LLaVA	0.77 → 0.48	0.43 → 0.22
UTKFace	QGFace-LLaVA + CMRR	0.78 → 0.35	0.41 → 0.14

**Table 12:** Direct quantitative validation of the task-aware metadata quality score.

Dataset/Setting	Mean $q_{t}$, Reliable	Mean $q_{t}$, Unreliable	AUROC	Association/Effect Size (95% CI)	Holm-Adjusted *p*-Value
CelebA-40/natural gender correctness	0.59	0.50	0.72	$r_{rb}$ = 0.32 [0.22, 0.42]	0.013
UTKFace/natural age reliability	0.78	0.53	0.81	ρ = −0.46 [−0.50, −0.42]	0.011
CelebA-40/Noisy60 corruption status	0.63	0.47	0.81	ρ = −0.37 [−0.41, −0.33]	0.007
UTKFace/Noisy60 corruption status	0.79	0.46	0.88	ρ = −0.54 [−0.63, −0.46]	0.012

**Table 15:** Cross-seed metadata-control failure cases under naturally occurring MiVOLO age-estimation errors on UTKFace.

Case	Age (GT/MiVOLO)	Abs. Age Error	Base Pred.	Full Pred.	Quality Score $q_{t}$	Gate Magnitude	Failure Mode
1	26/48	22	29.7 ± 1.1	35.1 ± 0.9	0.731 ± 0.027	0.475 ± 0.033	Reliability overestimation
2	62/43	19	58.3 ± 0.8	52.5 ± 1.5	0.696 ± 0.031	0.414 ± 0.027	Metadata-induced directional shift
3	19/39	20	23.3 ± 1.1	27.3 ± 1.3	0.672 ± 0.025	0.561 ± 0.043	Insufficient gate suppression
4	48/31	17	48.2 ± 1.4	53.7 ± 1.2	0.630 ± 0.036	0.423 ± 0.037	Adverse residual interaction

**Table 16:** Metadata utility boundary analysis based on clean-setting gain and semantic-mismatch sensitivity.

Dataset	Method	Clean Gain ↑	MDI ↓
CelebA-40	Naive Fusion	+0.11	0.250
CelebA-40	QGFace-LLaVA	+0.63	0.050
CelebA-40	QGFace-LLaVA + CMRR	+0.67	0.020
UTKFace	Naive Fusion	+0.475	0.401
UTKFace	Gate Only	+3.172	5.421
UTKFace	QGFace-LLaVA	+1.514	0.323
UTKFace	QGFace-LLaVA + CMRR	+1.548	0.152

Note: ↑ indicates that higher values are better, whereas ↓ indicates that lower values are better.

## Data Availability

The publicly available datasets used in this study are available from their official sources. FER2013 [12] is available at https://www.kaggle.com/c/challenges-in-representation-learning-facial-expression-recognition-challenge/data (accessed on 9 August 2026). CelebA/CelebA-40 [2] is available at https://mmlab.ie.cuhk.edu.hk/projects/CelebA.html (accessed on 9 August 2026). UTKFace [3] is available at https://susanqq.github.io/UTKFace/ (accessed on 9 August 2026).The source code developed for this study, dataset organization instructions, and summary experimental results are available at https://github.com/fjp181301-design/QGFace-LLaVA-official (accessed on 9 August 2026).

## Abbreviations

The following abbreviations are used in this manuscript:

MLLM	Multimodal large language model
LLM	Large language model
CMRR	Counterfactual metadata reliability regularization
MDI	Metadata Dependence Index
FER	Facial expression recognition
mAcc	Mean attribute accuracy
MAE	Mean absolute error
